# Restoring brain functions in the Mo-blo mouse model of Menkes disease with copper nanoclusters

**DOI:** 10.1126/sciadv.aeg6972

**Published:** 2026-09-30

**Authors:** Audrey Malardé, Jean-Marc Alberto, Benjamin Creusot, Laure Brice, Justine Paoli, Amélia Julien, Rémy Umoret, Pauline Renard, Grégory Pourié, Hervé Sartelet, Philippe Arnoux, Florent Barbault, Eric Brémond, Emma Jung-Rodriguez, Alexa Courty, Michael Walls, Luiz Tizei, Bruno Madebène, Etienne Derat, Benoit Tremblay, Ali Ouadi, David Brasse, David Coelho, Arnaud Wiedemann, David Meyre, Igor Clarot, François Feillet, Ariane Boudier

**Affiliations:** ^1^ Université de Lorraine, Inserm, NGERE, F-54000 Nancy, France.; ^2^ Université de Lorraine, CITHEFOR, F-54000 Nancy, France.; ^3^ Université de Lorraine, CNRS, LRGP, F-54000 Nancy, France.; ^4^ Department of Pathology, Nancy Regional University Hospital, F-54000 Nancy, France.; ^5^ Université Paris Cité, ITODYS, CNRS, F-75013 Paris, France.; ^6^ Sorbonne Université, MONARIS, CNRS-UMR 8233, F-75005 Paris, France.; ^7^ Laboratoire de Physique des Solides, Université Paris-Saclay, F-91405 Orsay, France.; ^8^ Sorbonne Université, IPCM, CNRS-UMR 8232, F-75005 Paris, France.; ^9^ Institut Pluridisciplinaire Hubert Curien, Université de Strasbourg, CNRS, UMR 7178, F-67037 Strasbourg, France.; ^10^ Pediatric Department Reference Center for Inborn Errors of Metabolism Children University Hospital Nancy, F-54000 Nancy, France.; ^11^ Nancy University Hospital, Department of Molecular Medicine, Division of Biochemistry, Molecular Biology, and Nutrition, F-54000 Nancy, France.; ^12^ Institut Universitaire de France (IUF), Paris, France.

## Abstract

Menkes disease, caused by *ATP7A* mutations, leads to severe copper deficiency and fatal neurodegeneration. Current copper-histidine therapy fails to restore brain function. Copper nanoclusters (CuNCs) were designed for their ultrasmall size to cross physiological barriers and deliver copper directly to the whole organism, including the brain. In *Mo-blo* mice (ATP7A deficient), daily subcutaneous CuNCs markedly improved survival, normalized cuproprotein activities, and restored cognitive and motor functions. Unlike other treatments, CuNCs effectively penetrate the blood-brain barrier, deliver copper to cuproproteins, offering unprecedented neurological rescue. This breakthrough could transform Menkes disease treatment, providing a first-ever therapy for brain copper deficiency. Further clinical development may unlock lifesaving solutions for this rare, fatal disorder.

## INTRODUCTION

Menkes disease (MD), also known as Menkes syndrome or kinky hair disease is an X-linked disorder affecting 1 of 300,000 male newborns in Europe (*1*, *2*). MD’s clinical presentation includes kinky hair, hypopigmentation, connective tissue defects, and severe neurodegeneration with a fatal outcome in early childhood (*3*). MD is due to a copper transporter deficiency (ATP7A), localized mainly in the gut and the blood-brain barrier (BBB) for copper transport in the blood, brain, and peripheral organs such as the kidneys and the liver (*4*). The dysfunction of copper transport arising from *ATP7A* mutation leads to accumulation of copper in the intestine due to failure to absorb this element and results in copper deficiency in the whole body, including the brain.

This copper deficit, consequently, induces reduced activities of all copper-dependent enzymes, such as cytochrome c oxidase (COX), superoxide dismutase, lysyl oxidase (LOX), tyrosinase (TYR), hephaestin and hephaestin-like 1 (HEPHL1), dopamine β-hydroxylase (DBH), peptidyl-α-amidating enzyme, sulfhydryl oxidase, and histaminase (*5*). This, thus, leads to the severe multisystemic manifestations observed in patients with MD, including neurodegeneration and progressive neurological deterioration, vascular abnormalities and connective tissue defects, hypopigmentation, skeletal dysplasia, and failure to thrive (*3*, *6*). To date, the only available treatment is copper-histidine (CuHis) complex that allows peripheral MD improvement. However, due to a poor biodistribution to the brain, the fatal prognosis remains unchanged (*7*).

Current research and clinical trials for treating MD involve CuHis associated with cerebral gene therapy, an invasive and expensive method (*8*, *9*), or elesclomol, a Cu^2+^-based anticancer drug, which improves cerebral copper absorption (*10*). However, the use of elesclomol remains highly debated due to the occurrence of adverse effects, particularly cutaneous reactions. In addition, this compound may induce deleterious oxidative stress associated with cellular toxicity and tissue damage, potentially limiting its therapeutic applicability (*11*–*13*). So far, clinical trials have not yet begun, according to clinicaltrials.org. Despite attempts to improve copper brain distribution, treatments for the neurological MD symptoms are still needed.

Due to their small size, copper nanoparticles (CuNPs) diffuse in different organs, mainly in the liver, kidneys, and spleen for particles measuring between 23.5 nm and 17 μm (*14*). Very small NPs reach organs more easily by persorption (4 nm) (*15*), transcellular or paracellular uptakes (4 to 10 nm) (*16*), allowing physiological barriers to be crossed, by bypassing the physiological transporters (*15*). Depending on their design, some NPs can cross the BBB and treat the neurological disease’s symptoms (*17*). However, they usually present a large distribution with generally greater diameters (*18*), preventing their elimination and leading to possible toxicity. This led us to design copper nanoclusters (CuNCs) with a much smaller size than NPs to avoid these drawbacks to treat MD. Intermediary between molecules and NPs (i.e., <2 nm in diameter) (*19*), it has already been shown that NCs are perfectly defined and organized structures, with a fixed atom number and high purity (*20*). Kidneys can eliminate NCs without inducing any immune response (*21*). These elements represent substantial advantages for potential medical applications compared with NPs even if none of them have ever been tested to treat MD so far. The use of CuNCs for nanomedicine is quite new (∼20 years), but these objects seem highly relevant for biomedical applications thanks to their unique properties as illustrated by recent reviews (*22*–*24*). Most research focuses on the noble metal NCs, but none of them have ever reached the clinical level. For instance, researchers have proved their performance for cancer imaging or treatment using gold NCs with radiotherapy (*18*).

The CuNCs that we present here can cross all barriers, including the BBB, due to their size, to deliver copper to apoproteins and restore their enzymatic functions at the peripheral and central level (Fig. 1). The innovation relies on an optimized and simple soft synthesis process based on the universal “Keep It Simple, Stupid” (KISS) principle, perfectly adapted for the future industrial scale-up and regulatory file (*25*). It is achieved by using selected biocompatible and bio-assimilable biomolecules, with chosen properties as the reductant and/or ligand. Consequently, CuNCs were synthesized according to this KISS process with limited synthesis steps, in water, and using raw materials such as histidine, ascorbic acid, and copper salt. Because these raw materials do not induce metabolic disorders, they were chosen to be consistent with medical use. Not only does this fit with nanomedicine, but it is also completely different from previously studied NCs (*20*, *26*). In this work, the developed CuNCs were tested in Mo-blo mice, an animal model of MD that displays a phenotype more closely resembling the human clinical presentation than other animal models (*27*–*29*). Similar to affected patients, Mo-blo mice exhibit altered hair color and fragility, reduced skin elasticity, and cardiovascular defects, as well as motor and cognitive deficits. The cardiovascular abnormalities, directly associated with connective tissue alteration, are characteristics not observed in the other murine models. After repeated subcutaneous injections, biochemical parameters were improved with normalization of cuproprotein activities. CuNCs were able to reach the central nervous system (CNS), and the neurological symptoms were strongly and significantly improved.

**Fig. 1.**
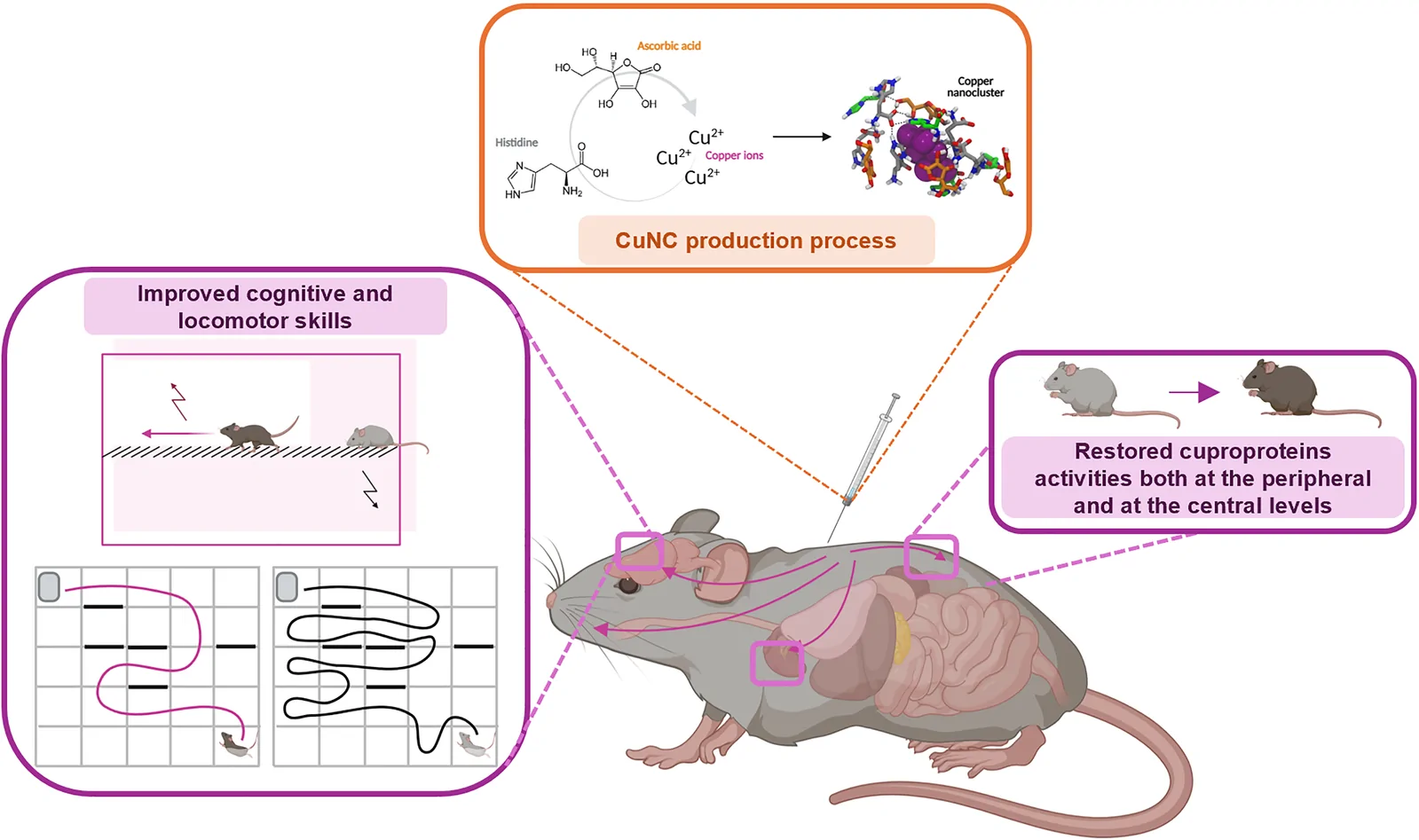
Therapeutic strategy using CuNCs for MD. Biocompatible CuNCs synthesized via sustainable green chemistry exhibit efficient trans-barrier transport in Mo-blo murine models. Following cellular uptake, CuNCs facilitate controlled intracellular copper release, enabling incorporation into apo-cuproproteins and restoration of their enzymatic activity. This targeted delivery mechanism results in significant improvement of both biochemical deficits and neurological dysfunctions characteristic of MD. Created in BioRender. Audrey, M. (2026), https://BioRender.com/mcldxkz.

## RESULTS

### CuNC molecular architecture and three-dimensional structuration

The controlled reduction of the CuHis complex by ascorbic acid led to CuNC formation. This is a soft process compared with other conditions described in the literature (*30*) and is well adapted to the application and the future regulatory issues to be addressed. Copper reduction and incorporation in NCs were of 100.5 ± 2.7% as evaluated by inductively coupled plasma–optical emission spectroscopy (ICP-OES) after NC purification by size exclusion chromatography (SEC). The NC scale was confirmed by the absence of plasmonic band and the presence of innate fluorescence (excitation wavelength of 379 ± 1 nm and emission wavelength of 457 ± 1 nm; Fig. 2, A and B). This result demonstrated the presence of NCs with ≤13 copper atoms (*31*, *32*) and the absence of nanoparticles or aggregates. Only the ultraviolet (UV) bands of histidinate and ascorbate were observed at 210 and 265 nm, respectively. Because no signal broadening was observed by nuclear magnetic resonance (NMR) (fig. S1), no radical was formed (*33*). This was compatible with cryo–scanning transmission electron microscopy (cryo-STEM) combined with electron energy-loss spectroscopy (EELS) studies, in which the composition was screened (Fig. 2, C and D). As a result, Cu(0) redox state was confirmed (*26*). CuO traces were measured at the surface of some particles that were attributed to the ligand binding (histidinate, ascorbate, or acetate). Based on infrared spectra (Fig. 2E), the interaction between the copper core and the ligands was proved via carbonyl (ascorbate), carboxylate (histidinate and acetate), and imidazole (histidinate) groups. The observed band shifts of ascorbate (C═O band at 1753 cm^−1^ shifted in two bands in CuNCs and C═C band at 1655 cm^−1^ shifted at 1640 cm^−1^ in CuNCs) were consistent with its interaction with CuNCs (*34*, *35*). For histidine and acetate, the asymmetric and symmetric stretching modes, ν_as_(COO^−^) and ν_s_(COO^−^) (*36*), were observed at 1560 and 1410 cm^−1^, respectively. For CuNCs, these bands were observed at a very similar position [at 1510 (shoulder) and 1347 cm^−1^] and were consistent with the interaction of carboxylate groups with the CuNCs (*37*). For histidine, the N─H bending observed at 1140 cm^−1^ was found at 1155 cm^−1^ in CuNCs, proving its interaction with CuNCs (*38*) via the imidazole group also confirmed by ^1^ H and ^13^C NMR analyses (fig. S1). NCs were noncharged as observed by capillary zone electrophoresis (Fig. 2F) as their migration time was the same than the one of the neutral marker (N), benzyl alcohol.

**Fig. 2.**
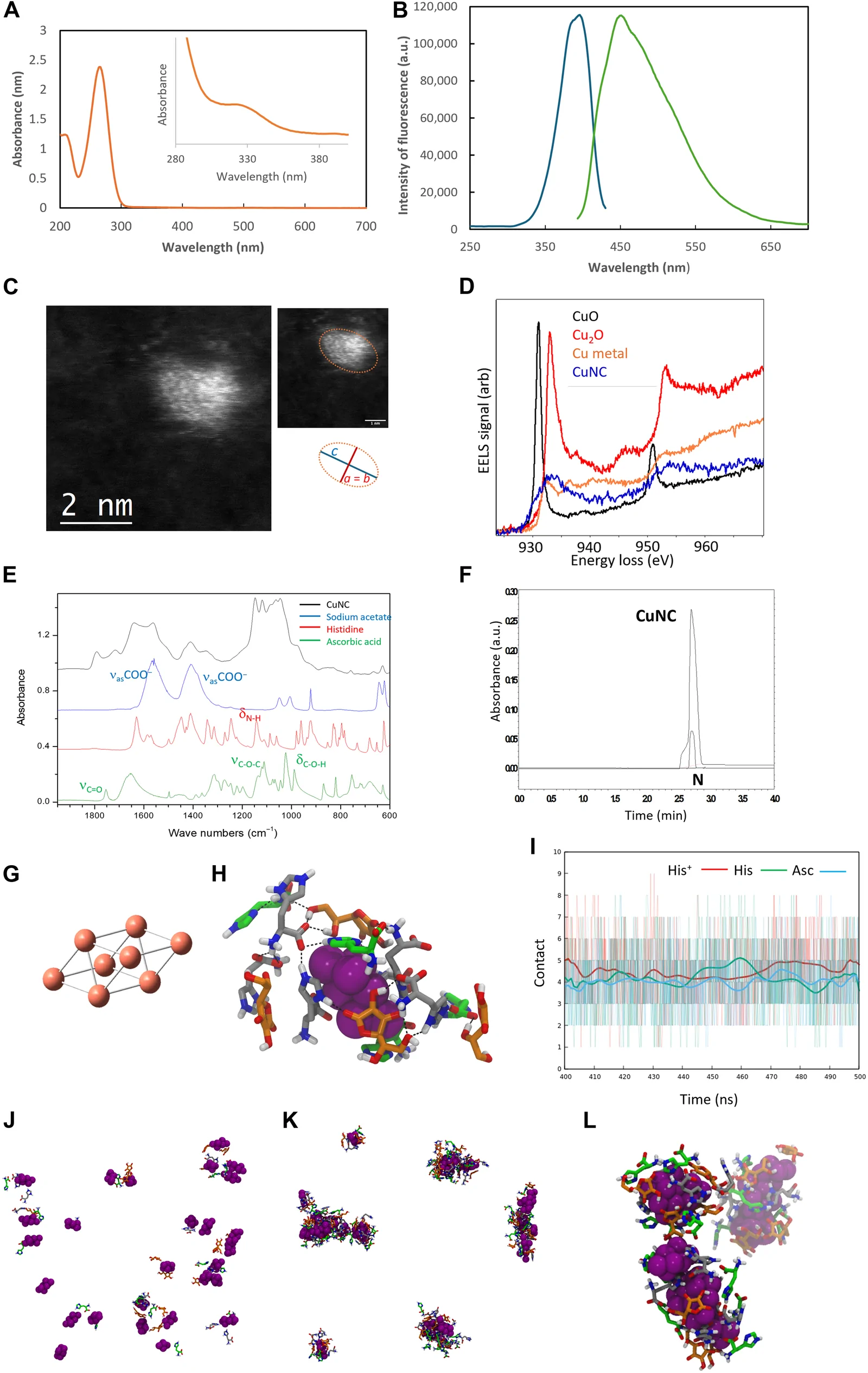
CuNC characterization. (**A**) UV-visible at 0.25 mg/liter of copper; inset graph is a zoom between 300 and 400 nm at 7.8 mg/liter of copper. (**B**) Excitation (blue) and emission (green) fluorescence spectra. (**C**) Typical observation of CuNCs using cryo–transmission electron microscopy (TEM) equipped with high-angle annular dark field (HAADF) and the method used to measure the dimensions of auto-associated objects and (**D**) analysis of CuNCs by cryo-TEM equipped by electron energy-loss spectroscopy (EELS). (**E**) Fourier transformed infrared spectra of CuNCs. (**F**) Typical electropherogram of CuNC; N stands for neutral species, i.e., neutral marker, benzyl alcohol. CuNCs modeled by (**G**) density functional theory (DFT) and (**H**) molecular dynamics (MoD) typical visualization of CuNC association with histidinate and ascorbate residues; CuNC is displayed with van der Waals spheres in purple while histidinate and ascorbate residues are represented by sticks. Protonated and neutral histidinate residues have their carbons colored in gray and green, respectively, and ascorbate has its carbons in orange. Only polar hydrogens are displayed, and dashed lines represent hydrogen bonds. (**I**) Example of several residues interacting with CuNCs along the last 100 ns of MoD trajectory. (**J** and **K**) Example of auto-association processes: the CuNC structures (purple) surrounded by histidinate and ascorbate residues at the beginning and the end of MoD (duration of 1 μs). (**L**) Example of shape found in the aggregates for the MoD simulations.

Based on Fermi’s theory of magic numbers for atoms in NCs leading to stable and fluorescent NCs (*39*, *40*), we have considered an eight-atom copper core Cu_8_NC. Density functional theory (DFT) confirmed the improved stability of this stoichiometry (Fig. 2G) by showing an increase of the highest occupied molecular orbital–lowest unoccupied molecular orbital gap for this size (see the Supplementary Materials for the detailed study). Molecular dynamics (MoD) revealed different associations between the metallic core and organic ligands (histidinate and ascorbate, see the Supplementary Materials for the detailed study). The copper core is permanently covered by ligands in a dynamic process: ligands being exchanged around the NC while maintaining a substantial interaction number, demonstrating that the NC had a strong affinity for histidinate and ascorbate (Fig. 2, H and I, and movies S1 and S2). Numerous van der Waals interactions were observed between the ligands and the NC. Last, stabilizing interactions, i.e., mainly electrostatic and hydrogen bonds, were established among ligands, involving the positively charged ammonium groups of the amino acid backbones (N terminus) and the protonated imidazole rings of histidinate residues (His^+^) with the negatively charged ascorbate regions and the carboxylate group of the amino acid backbone (C terminus). Based on MoD comparisons with and without ascorbate (see the Supplementary Materials), this molecule did not significantly reduce the histidinate number around NC, suggesting a stabilization of the NC and highlighting the essential role of ascorbate in CuNCs. The MoD data gave a generic formula of Cu_8_Histidinate_8.7 ± 0.8_Ascorbate_4.0 ± 0.5_, compared with Cu_8_Histidinate_14.5 ± 1.0_Ascorbate_1.5 ± 0.8_Acetate_0.2 ± 0.1_ [obtained by high-performance liquid chromatography (HPLC)–UV after purification by SEC]. The MoD approach was brought a further step to explore the possibility of an association involving several CuNC (*41*). After 1 μs, irregular ellipsoid auto-associations of 8 to 10 CuNCs were observed. They were amplified by ascorbate that established long-range electrostatic interactions with the cationic histidinate parts, especially those with His^+^, thereby facilitating different CuNC associations (Fig. 2, J and K, and movies S3 and S4). The average dimension of eight auto-associated CuNCs was on the short lengths, *a* = *b* = 0.7 nm, and *c* = 2.0 nm on the long one. Cryo-STEM equipped with high-angle annular dark field (HAADF) showed the presence of irregular quasi-ellipsoidal structures with diameter dimensions of *a* = *b* = 0.8 ± 0.2 nm and *c* = 1.7 ± 0.4 nm (evaluated on 23 CuNCs, as illustrated in Fig. 2C). In addition, structures of hydrodynamic diameters equal to 2.1 ± 0.1 nm were also measured by dynamic light scattering (DLS; fig. S2). At the lowest concentrations, for example, after dilution in purified water or in phosphate-buffered saline (PBS), these auto-associations disassembled in smallest structures measured at 0.7 ± 0.1 nm (hydrodynamic diameters obtained by DLS), which remained stable during 6 hours. CuNCs were characterized by high storage stability (>2 years; fig. S3), using DLS to screen the presence of aggregates, which was both interesting and unexpected (*42*), emphasizing once again the essential role of ascorbate.

### Modeling of CuNC ability to cross the BBB and to deliver copper

MoD simulations were performed to investigate the interaction of the CuNC with a model of the BBB membrane (*43*). The constructed lipid bilayer remained structurally stable throughout equilibration, confirming the reliability of the model (Fig. 3). The simulations revealed that CuNC approaches the membrane surface, where its histidine and ascorbate ligands gradually dissociate. These forms stabilize interactions between the polar headgroups of lipids and the CuNC. This process facilitates the embedding of the CuNC core within the hydrophobic region of the bilayer, without inducing detectable perturbations of the membrane structure. Density and distribution analyses indicated that only the Cu_8_ species penetrates the membrane, while the histidine and ascorbate ligands remain in the aqueous phase, highlighting a kind of vectorization role. No complete translocation of Cu_8_ across the bilayer was observed within the accessible microsecond timescale. However, to go further in this investigation, DFT calculations indicated that Cu_8_NCs are markedly more stable in polar than in nonpolar environments, suggesting a destabilization within the hydrophobic core of lipid membranes. This effect may promote structural distortion and partial embedding of Cu_8_ in the bilayer. We therefore hypothesized that the key step in copper distribution is the insertion of the NC alone into the lipid core of the membrane, where it becomes destabilized to lose its structural integrity, thereby enabling the release and distribution of copper within the cell, allowing its incorporation in cuproproteins. Full methodological details and additional analyses are provided in the Supplementary Materials and movies S5 and S6.

**Fig. 3.**
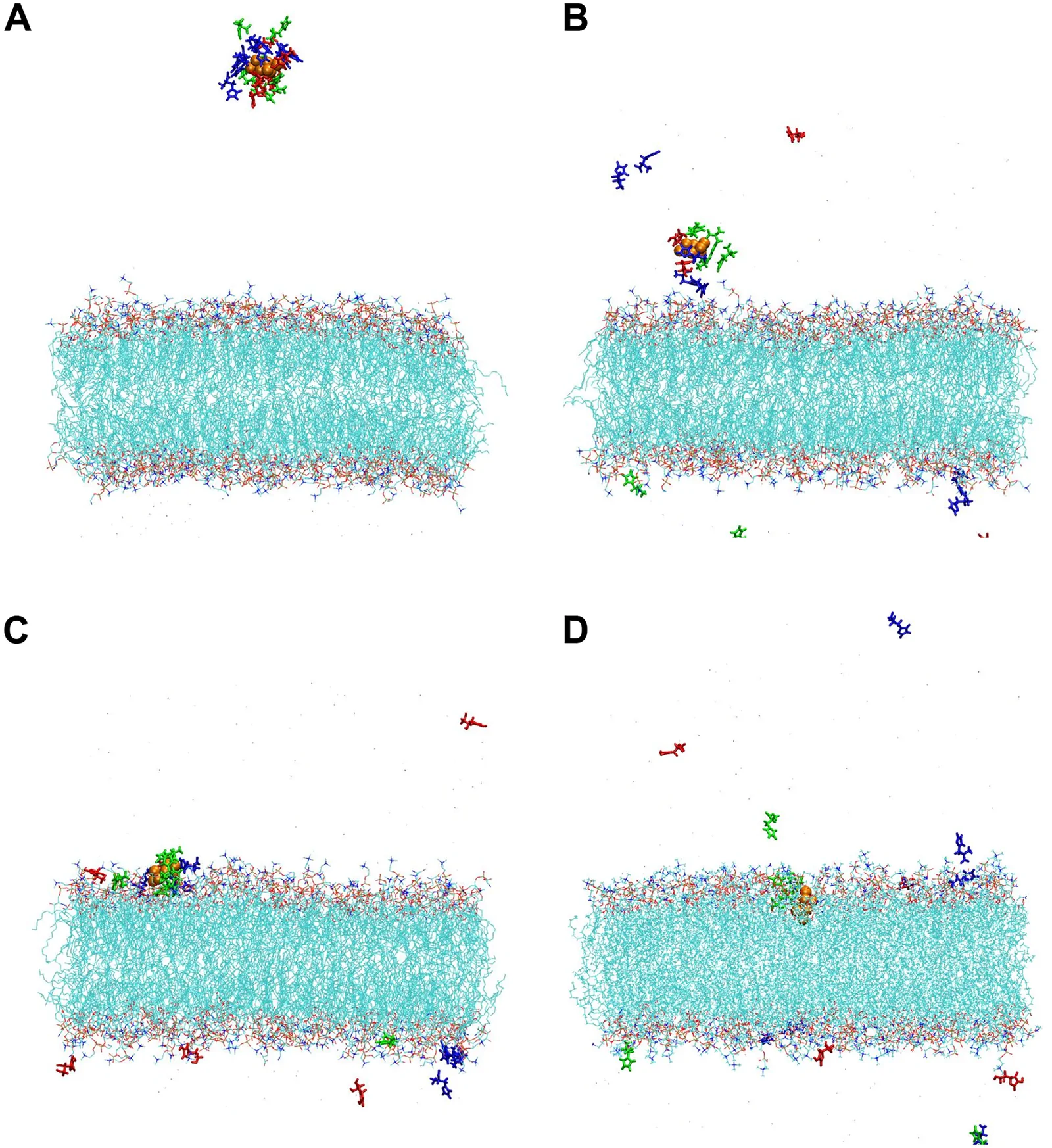
CuNCs reaching the BBB membrane obtained by MoD simulation. Calculations during 100 ns [beginning (**A**) and end (**B**)] and 10 μs starting after the first simulation [beginning (**C**) and end (**D**)]. Ligands appearing below the membrane belong to the other periodic box situated below the presented system. Typical movies are presented in the Supplementary Materials.

### Mo-blo model presentation

Mo-blo mice (*Atp7a*^−^) exhibited a typical MD phenotype closely mirroring that observed in affected children. In mice, this was manifested by light-colored fur and curly whiskers, directly reflecting patient features such as hypopigmented hair and pili torti (Fig. 4, A and B, and the Supplementary Materials) (*44*, *45*). These mice also showed markedly reduced *Atp7a* mRNA expression due to decreased transcription of corresponding gene (Fig. 4C), as well as a loss of ceruloplasmin expression and enzymatic activity (Fig. 4, D and E). This genetic alteration then led to defective systemic copper transport, resulting in the observed clinical manifestations. The hair phenotype resulted from diminished expression and activity of TYR, a copper-dependent enzyme, is essential for melanin synthesis via the pyrrole-2,3,5-tricarboxylic acid (PTCA) precursors (Fig. 4, F and G), as well as from impaired activity of copper-dependent pathway involved in hair integrity. In particular, functional deficiency of HEPHL1, a ferroxidase implicated in epithelial copper and iron homeostasis, may indirectly contribute to abnormal hair and vibrissae structure by altering copper availability required for LOX activity in keratinocyte-associated extracellular matrix organization (Fig. 4B). Mo-blo mice had a median in survival of 23 days (*n* = 14) (Fig. 4H) and postmortem analyses revealed that death primarily resulted from marked hemorrhage caused by aortic aneurysm (Fig. 4I). This vascular defect was explained by an altered activity of LOX activity, inducing default in collagen and elastin integrity, despite unaltered intracellular and secreted LOX expression (Fig. 4, J and K, and the Supplementary Materials). Neurologically, Mo-blo mice exhibited significantly decreased expression in the cerebral cortex and activity in the striatum of COX (complex IV of the mitochondrial respiratory chain), resulting in impaired adenosine 5′-triphosphate production and increased oxidative stress in neuronal tissues (Fig. 4, M and N). This mitochondrial dysfunction contributed to severe neurological deficits in the mice, including motor coordination impairments and cognitive dysfunctions.

**Fig. 4.**
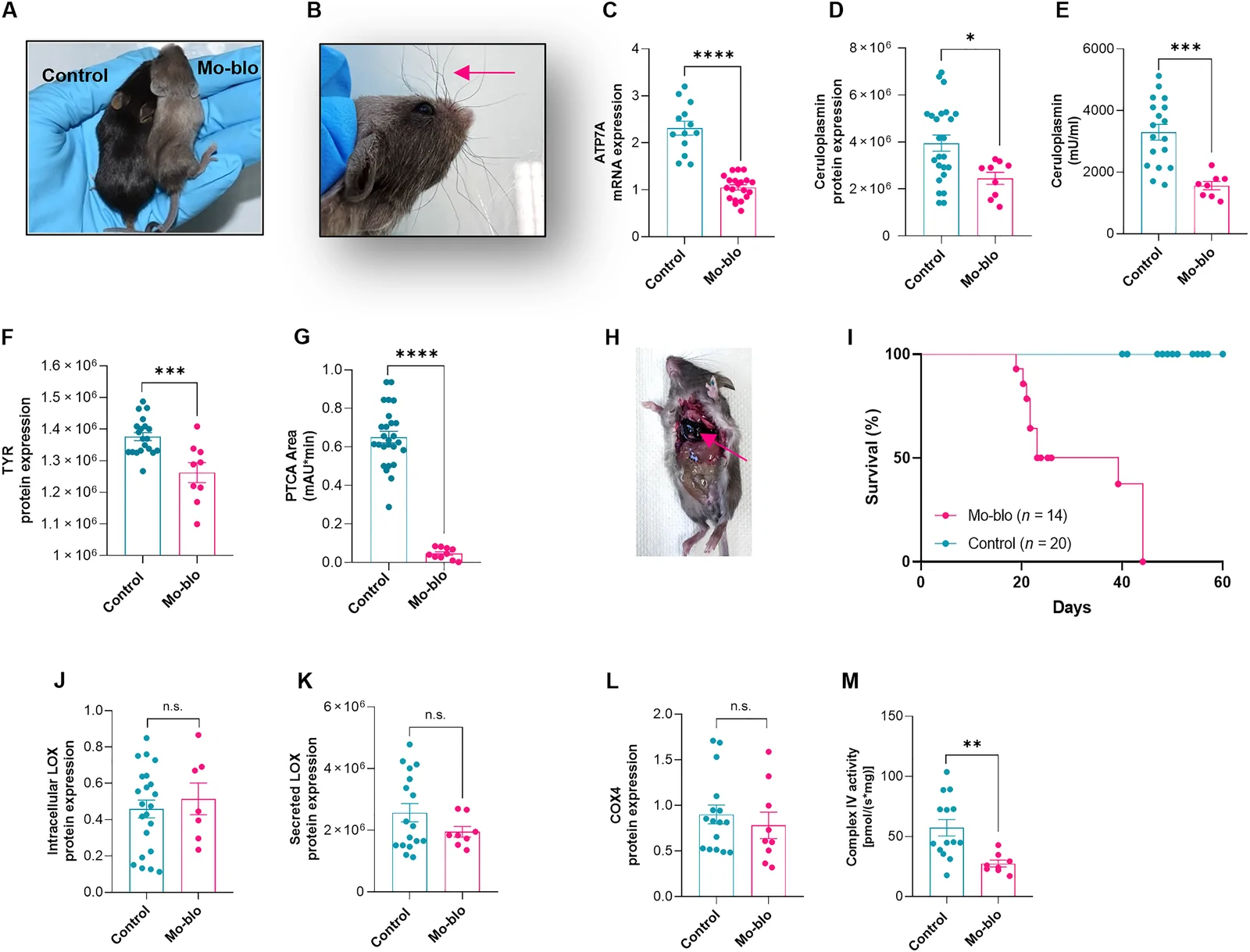
Phenotypic and molecular characterization of Mo-blo mice (Mo-blo) versus wild-type mice (control). (**A**) Representative images showing a Mo-blo mouse with light coat color (right) compared with a wild-type littermate with black fur (left) [at postnatal day 15 (PND15)]. (**B**) Mo-blo mice display abnormally coiled whiskers (➔). (**C**) Relative expression of Atp7a mRNA in brain tissue from Mo-blo versus wild-type mice, as measured by reverse transcription quantitative polymerase chain reaction (RT-qPCR; control, *n* = 13; Mo-blo, *n* = 20). (**D** and **E**) Ceruloplasmin protein levels and ferroxidase activity in plasma from PND21 mice assessed by Western blot (control, *n* = 24; and Mo-blo, *n* = 9) and colorimetric assay (control, *n* = 18; and Mo-blo, *n* = 8), respectively. (**F** and **G**) TYR protein levels (control, *n* = 20; and Mo-blo, *n* = 9) and melanin (via PTCA quantification) content (control, *n* = 26; and Mo-blo, *n* = 10) in skin and hair from PND21 mice, analyzed by Simple Western and HPLC. (**H**) Macroscopic image of a PND21 Mo-blo mouse after death due to an aortic aneurysm. (**I**) Kaplan-Meier survival curve showing increased mortality in Mo-blo mice (control, *n* = 20; and Mo-blo, *n* = 14). (**J** and **K**) Tissue (heart) (control, *n* = 22; and Mo-blo, *n* = 7) and circulating (plasma) (control, *n* = 17; and Mo-blo, *n* = 8) LOX protein levels in PND21 mice analyzed by Simple Western and Western blot, respectively. (**L** and **M**) COX protein levels (control, *n* = 16; and Mo-blo, *n* = 9) and complex IV activity (control, *n* = 14; and Mo-blo, *n* = 9) in the brain of PND21 mice, assessed by Western blot and high-resolution respirometry (or oxygraphy). Data are presented as means ± SEM. Statistical significance was determined by unpaired *t* test and is indicated as follows: *P < 0.05; **P < 0.01; ***P < 0.001; ****P < 0.0001. n.s., not significant.

### CuNC biodistribution to the filter organs and the brain

CuNCs were administrated to Mo*-*blo mice by subcutaneous injections (Fig. 5 and the Supplementary Materials) from postnatal day 5 (PND5) to PND25. Copper biodistribution was compared across CuHis, CuNCs, and a wild-type (control) (at PND26) group using positron emission tomography (PET) at 4 and 24 hours postinjection. For labeling, ^64^Cu was incorporated into ^64^CuNCs, ^64^CuHis or ^64^Cu-acetate for the control group. Following imaging, organs were collected to quantify the radioactivity amount and determine the copper biodistribution patterns.

**Fig. 5.**
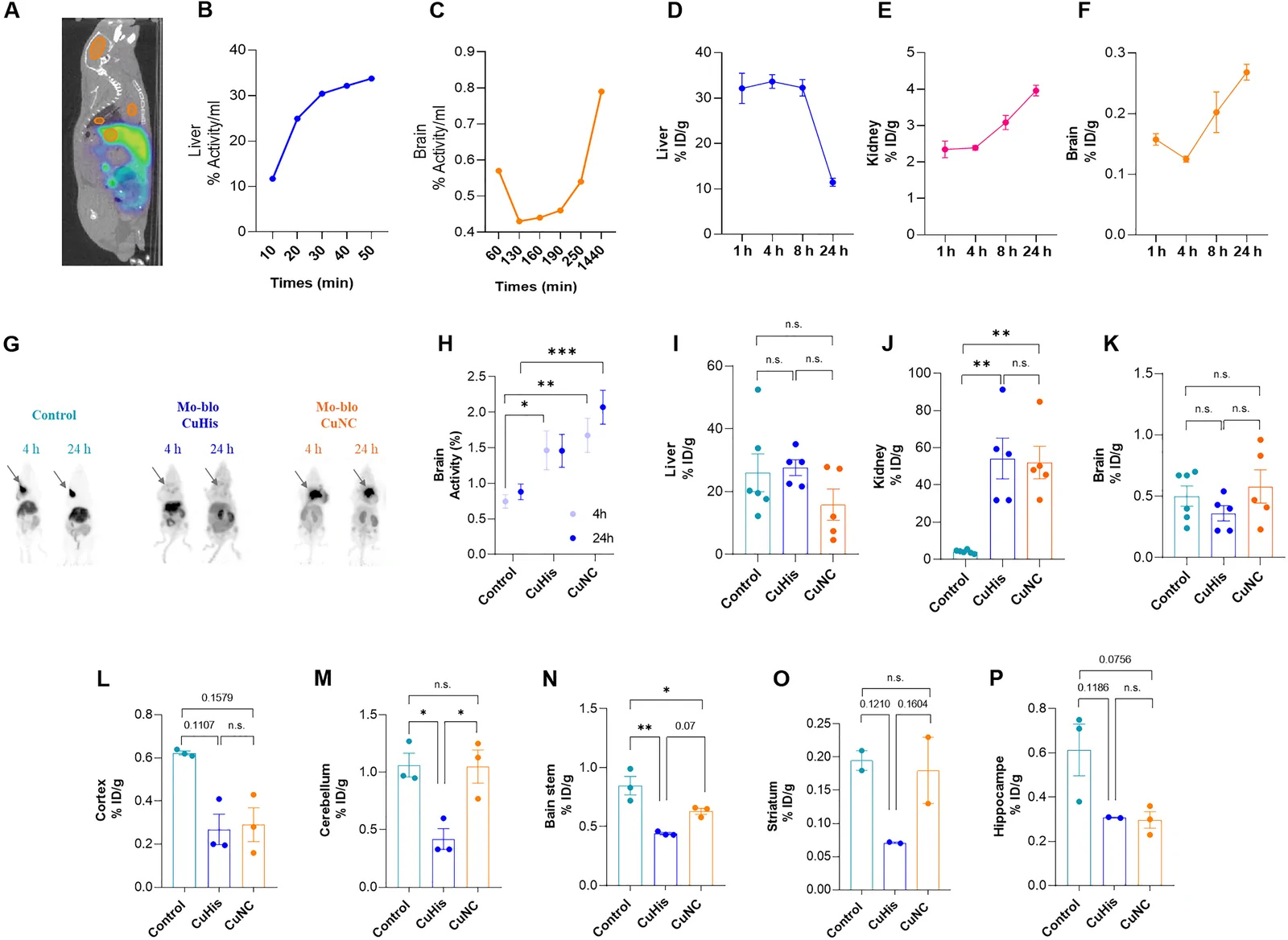
Biodistribution of ^64^Cu assessed by positron emission tomography (PET) at PND26. (**A**) Preliminary evaluation of ^64^CuNC biodistribution in wild-type. (**B** and **C**) In vivo time-course quantification of ^64^CuNCs uptake in the liver (*n* = 1) and brain (one mouse per time point) from 1 to 24 hours. (**D** to **F**) Ex vivo quantification of ^64^Cu accumulation in major filtering organs (liver and kidney) and brain at 1, 4, 8, and 24 hours (h; *n* = 4 for 1, 4, and 8 hours; and *n* = 6 for 24 hours) in wild-type mice. (**G**) In vivo visualization of ^64^Cu distribution in Mo-blo mice, the arrow indicates the site of injection. (**H**) In vivo quantification of ^64^Cu signal in the brain of Mo-blo mice treated with CuHis (*n* = 4) or CuNCs (*n* = 5), compared with wild-type (control; *n* = 6) from 4 to 24 hours. (**I** to **K**) Ex vivo biodistribution of ^64^Cu in filtering organs (liver and kidney) and brain at 24 hours in Mo-blo mice treated with CuNCs (*n* = 5) or CuHis (*n* = 5), compared with wild-type mice treated with copper acetate (control; *n* = 6). (**L** to **P**) Ex vivo quantification of ^64^Cu in distinct brain regions associated with cognitive and locomotor functions (cortex, cerebellum, brainstem, striatum, and hippocampus) at 24 hours in control (*n* = 3 or *n* = 2), CuHis (*n* = 3 or *n* = 2), and CuNCs (*n* = 3 or *n* = 2) groups. Data represent means ± SEM. Statistical significance was determined using Welch’s analysis of variance (ANOVA) followed by Games-Howell’s post hoc test or using one-way ANOVA followed by Tukey’s test. n.s., not significant (*P* > 0.05); *P < 0.05; **P < 0.01; ***P < 0.001. ID, injected dose.

A preliminary study in a wild-type mouse injected with ^64^CuNCs established an initial distribution profile and optimal analysis time points (Fig. 5, A to C). This study was followed by a full study in the animals of interest, i.e., Mo-blo mice (Fig. 5, D to F).

Preliminary PET analyses showed that ^64^CuNCs primarily accumulated in filtering organs and were mainly excreted via the biliary tract with minimal renal elimination (Fig. 5, B, D, and E), consistent with known copper metabolism. A small fraction of the injected dose (ID) reached the brain exhibiting increased uptake from 1 to 24 hours postinjection (Fig. 5, C and F).

In a second series of experiments, Mo-blo mice were injected with ^64^CuNCs (CuNC), and in vivo PET data acquired at 4 and/or 24 hours (Fig. 5, G and H) were compared with those obtained from ^64^CuHis-treated (CuHis) and wild-type mice injected with ^64^Cu-acetate (control). These measurements were performed in vivo in anesthetized animals with intact blood circulation, enabling dynamic assessment of radiolabeled copper biodistribution under controlled physiological conditions. In Mo-blo mice brain, PET signal intensity was significantly higher following ^64^CuNC administration compared with controls at both 4 and 24 hours (Fig. 5H), suggesting enhanced and sustained accumulation of the copper-associated signal in the CuNC-treated group under controlled systemic conditions. For peripheral organs, in vivo PET and ex vivo analyses revealed too pronounced renal copper accumulation in Mo-blo mice regardless of treatment (>50% ID/g versus <5% ID/g in control), along with reduced hepatic uptake in CuNC-treated animals (∼16% ID/g) compared with CuHis (∼30% ID/g) (Fig. 5, G to J). This abnormal distribution arises from defective ATP7A-dependent copper trafficking leading to impaired hepatic copper incorporation (*46*) and pathological sequestration within renal cortical cells due to disrupted efflux pathway (*29*).

Regarding ex vivo cerebral copper distribution, no significant difference in total copper level (expressed as ID/g) was observed in Fig. 5K due to the difficulty in dissociating copper sequestered within the intravascular compartment from that present in the brain. These findings differed from the in vivo PET results shown in Fig. 5H, despite being obtained from the same animals, where ^64^CuNC administration produced a significant increase in cerebral PET signal. This discrepancy is likely attributable to methodological limitations inherent to PET imaging, including extracerebral radioactive activity, particularly at the injection site, as well as the limited spatial resolution of PET, leading to partial-volume effects that may affect accurate quantification of brain signal.

However, and notably, analysis of different brain regions (Fig. 5, L to P) performed on microdissected tissues, thereby limiting the contribution of residual intravascular copper, revealed significantly greater ^64^CuNC accumulation than ^64^CuHis in the brain stem, cerebellum, and striatum. In the latter two regions, which are highly dependent on energy consumption, ^64^CuNC uptake even approached levels observed in control, suggesting enhanced cerebral copper delivery and regional bioavailability.

Collectively, these findings suggest that CuNC treatment promotes more efficient copper transport across the brain barriers and enhances its regional distribution within the brain parenchyma. However, the functional consequences of this increased cerebral copper bioavailability remain to be established through complementary studies, including assessment of cognitive and locomotor functions involving these brain regions.

### CuNCs improve the life span of Mo-blo mice

Survival analysis showed a marked improvement in life span for Mo-blo mice treated with CuNCs (equivalent copper, 64 μg/ml) compared with untreated or CuHis-treated groups (equivalent copper, 64 μg/ml) (Fig. 6A). Median survival was 23 days for untreated mice and 30 days for CuHis-treated animals (CuHis), whereas CuNC-treated (CuNC) and wild-type (control) mice reached 50 days (Fig. 6B). This significant increase in survival demonstrates the therapeutic efficacy of CuNCs in restoring copper homeostasis and mitigating the lethal systemic manifestations characteristic of MD. Notably, untreated or CuHis-treated Mo-blo mice ultimately died from aortic aneurysm rupture, a hallmark of the model reflecting vascular fragility induced by LOX deficiency (Fig. 4H). Consequently, for subsequent analyses conducted beyond the median survival time, only the CuNC-treated and control groups were included to ensure experimental consistency and biological relevance.

**Fig. 6.**
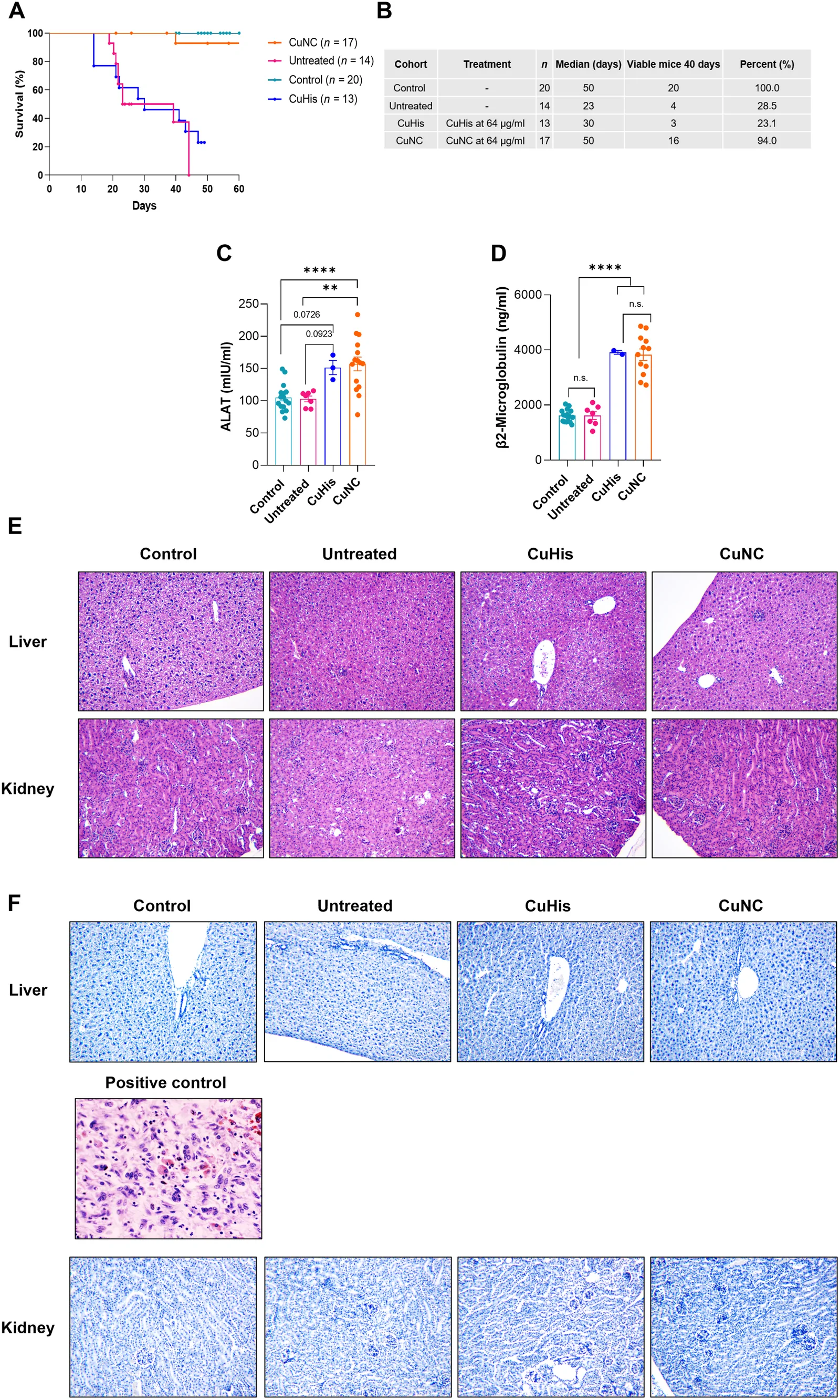
CuNC treatment extends life span. (**A**) Kaplan-Meier survival curves of Mo-blo mice treated with CuNCs (*n* = 17), untreated Mo-blo mice (untreated; *n* = 14), Mo-blo mice treated with CuHis (*n* = 13), and wild-type (control; *n* = 20). (**B**) Median survival of each group. (**C** and **D**) Hepatic and renal function assessed by ALT and β2-microglobulin levels in plasma using an ELISA test in the Mo-blo groups receiving no treatment (untreated; PND21, *n* = 7), Mo-blo treated with CuHis (PND50, *n* = 3 or *n* = 2), and Mo-blo treated with CuNCs (PND50, *n* = 15 or *n* = 12) compared to wild-type (control; PND50, *n* = 16) mice. ALAT, alanine aminotransferase. (**E** and **F**) Histopathological analysis of liver and kidney from Mo-blo untreated (Untreated, PND21), Mo-blo treated with CuHis (PND50) and CuNC-treated Mo-blo (PND50) mice, including copper-specific staining (rhodanine; E) and hematoxylin-eosin-saffron (HES; F) staining. Positive control is a liver sample with copper accumulation of the Wilson’s disease type. Data represent means ± SEM. Statistical significance was determined using one-way ANOVA followed by Tukey’s test. n.s., not significant (*P* > 0.05); **P < 0.01; ****P < 0.0001.

From a safety perspective, no macroscopic abnormalities suggestive of local inflammation or necrosis were observed at the injection site. Biochemical analyses of plasma samples from mice aged 21 to 50 days, depending on the experimental groups, revealed significantly elevated alanine aminotransferase levels in both CuNC- and CuHis-treated mice (≈150 mIU/ml) compared with control and untreated mice (≈100 mIU/ml) (Fig. 6C), as well as a twofold increase in β2-microglobulin concentrations in the same groups (≈4000 ng/ml versus ≈2000 ng/ml in control and untreated mice) (Fig. 5D). These reversible elevations suggest transient hepatic and renal stress, likely linked to adaptive responses to copper repletion. In contrast to previous studies, reporting deleterious copper accumulation in renal tissue (notably tubular necrosis) (*8*, *9*), histopathological analyses of the liver and kidneys from this same mouse cohort revealed fully preserved tissue architecture, with minimal inflammatory infiltration, mainly focal in the liver and predominantly lymphocytic and with no signs of necrosis, inflammation, or fibrosis (Fig. 6E). Furthermore, rhodanine staining, commonly used to diagnose copper overload disorders such as Wilson disease, confirmed the absence of copper deposits in hepatic and renal tissues, supporting the noncumulative nature of CuNC-mediated copper delivery (Fig. 6F). This outcome is attributable to a precisely calibrated dosage aligned with physiological requirements (8 μg/7 days), in contrast to conventional CuHis supplementation or prior studies administering up to 20 μg (*8*, *9*). Accordingly, the renal biomarker β2-microglobulin, routinely used clinically to assess renal injury, constitutes a relevant surveillance indicator for potential copper tissue accumulation during supplementation. Any observed elevations remain reversible through adjustment of CuNC dosing or administration frequency, mirroring therapeutic titration strategies typically applied in patients with MD treated with CuHis.

### CuNC efficacy on nonneurologic cuproenzyme impairment

MD is characterized by the inability to absorb dietary copper captured in enterocytes, leading to profound copper deficiency and a combined deficit in all cuproenzyme activities, both at the neurological and nonneurological levels. The efficacy of CuNCs on nonneurological cuproenzymes was evaluated in PND50 mice by examining TYR activity, which affects the development and pigmentation of cutaneous appendages (*47*), as well as pathways involving HEPHL1, a ferroxidase involved in epithelial copper homeostasis and associated with normal hair and whisker morphology (*48*). Alteration in HEPHL1 may disrupt local copper availability required for LOX activity, a cuproenzyme essential for extracellular matrix cross-linking and proper maturation of collagen and elastin, thereby contributing to abnormalities in hair structure and connective tissues (*45*). Treatment with CuNCs restored the structure of hair and whiskers, as evidenced by the normal appearance of whiskers in Mo-blo mice (Fig. 7, A and B). TYR activity recovery was phenotypically confirmed by the return to normal coat pigmentation in Mo-blo mice treated with CuNCs (Fig. 7A). Western blot quantification in mice skin revealed significantly reduced TYR apoprotein synthesis in all Mo-blo mice, whereas hair microscopic observation confirmed the presence of melanin pigment in hair from CuNC-treated mice (Fig. 7, C and D). This finding was further validated by TYR activity analysis via melanin pigment (PTCA) quantification in mice hair (Fig. 7E).

**Fig. 7.**
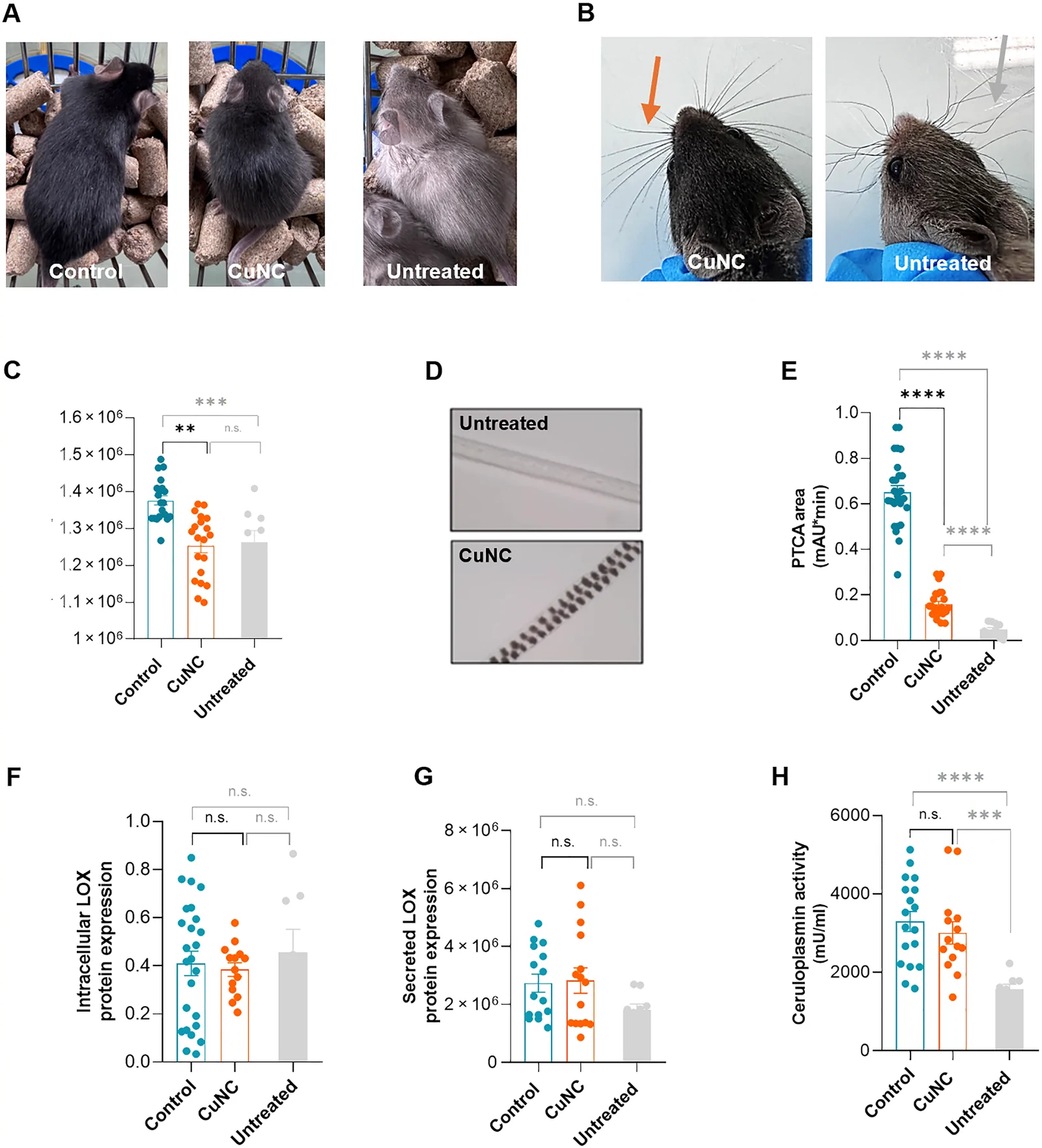
Efficacy of CuNC treatment on nonneurologic impairment (at PND50). (**A**) Phenotypic observations of coat and (**B**) whiskers (➔) of Mo-blo mice during treatment (CuNC) about the activity of the cupro-dependent proteins: HEPHL1, LOX, and TYR, respectively. Same picture as Fig. 4B used for comparison with CuNC-treated mice. (**C**) Quantification of TYR by Simple Western and (**D**) microscopic observations of melanin pigment production in the hairs (×40) in correlation with (**E**) the analysis of PTCA pigment, the result of enzymatic TYR activity. (**F** and **G**) Quantifications of LOX expression (intracellular and secreted forms) were performed by Simple Western and Western blot, respectively. (**H**) Quantifications of ceruloplasmin activity were performed by colorimetric assay. The shaded values for untreated group refer to the results shown in Fig. 4. TYR and PTCA analyses were carried out on skin and hairs samples using the following numbers: control, *n* = 20; CuNCs, *n* = 20; and control, *n* = 26; CuNCs, *n* = 26, respectively. Data are presented as means ± SEM. Statistical significance was calculated by one-way ANOVA with Tukey’s test and ANOVA Welch test following Games-Howell’s test. n.s., not significant (*P* > 0.05); ***P < 0.001; ****P < 0.0001. For LOX, analyses were carried out on heart (intracellular form) and plasma (secreted form), according to the following numbers: control, *n* = 25; CuNCs, *n* = 15; and control, *n* = 15; CuNCs, *n* = 15, respectively (biologically independent samples). Data are presented as means ± SEM. Statistical significance was calculated by ANOVA Welch test following Games-Howell’s and Kruskal-Wallis test with Dunn’s test. n.s., not significant (*P* > 0.05). Ceruloplasmin activity analyses were carried out on plasma samples using the following numbers: control, *n* = 18; and CuNCs, *n* = 14. Data are presented as means ± SEM. Statistical significance was calculated by one-way ANOVA with Tukey’s test and ANOVA Welch test following Games-Howell’s test. n.s., not significant (*P* > 0.05); **P < 0.01; ***P < 0.001; ****P < 0.0001.

Despite the restoration of animal survival and the absence of aortic aneurysms, the latter being caused by LOX deficiency, intracellular and secreted LOX expression levels in heart and plasma remained unchanged, indicating that correction of the nonneurological phenotype can occur independently of changes in this multicopper enzyme’s expression (Fig. 7, F and G).

Although ceruloplasmin is primarily a diagnostic marker, independent of copper trafficking by ATP7A, the Mo-blo model showed significantly reduced expression and activity of the protein (Fig. 4, D and E). Conversely, quantitative analysis of ceruloplasmin activity in plasma shows that mice treated with CuNC exhibit levels comparable with those of the control group, with no statistically significant difference (Fig. 7H). Because reduced ceruloplasmin activity is a biochemical characteristic of MD, these results indicate that CuNC treatment restores normal copper metabolism.

Overall, these results indicated that CuNC treatment effectively restores nonneurological cuproenzyme activity (mainly TYR, HEPHL1, and LOX), as demonstrated by both phenotypic improvements (coat pigmentation and whisker morphology) and biochemical parameters. Although apoprotein forms are present in Mo-blo mice, copper incorporation is essential for their enzymatic activity. The copper delivered by CuNCs appears available for proper incorporation, allowing holo-cuproenzyme formation and restoration of protein structure and function (*49*, *50*). To the best of our knowledge, this is the demonstration that CuNCs are able to deliver metal with this kind of cell trafficking, after subcutaneous administration.

### CuNC efficacy on MD neuronal damage

At the cerebral level, copper deprivation induces a COX (complex IV) deficiency, leading to a severe secondary impairment of the mitochondrial respiratory chain (*51*). When associated with reduced DBH activity (catecholaminergic neurotransmitter deficiency) and superoxide dismutase deficiency (prooxidant status), this metabolic triad contributes to the development of degenerative epilepsy encephalopathy, which is primarily responsible for locomotor and cognitive disorders (*52*). To evaluate the neuroprotective efficacy of CuNC treatment in Mo-blo mice aged 50 days, COX expression in the cortex and its activity in the striatum, an energy-demanding brain region particularly sensitive to copper-related mitochondrial dysfunctions, were analyzed. In parallel, the study of locomotor (at PND27 and PND28) and neurocognitive performance (learning and memory, from PND33 to PND38) associated with hippocampal neurodegeneration were evaluated (Fig. 8) (*53*). This combined biochemical and functional evaluation aimed to determine whether CuNC-mediated copper delivery could restore neuronal energy metabolism and improve neurological outcomes. The results showed that no significant difference in COX subunit protein expression was observed between wild-type (control) and CuNC-treated Mo-blo mice (CuNC) (Fig. 8A). However, complex IV activity was restored at a physiological level following CuNC treatment, reaching values comparable with those of control mice. (Fig. 8, B and C).

**Fig. 8.**
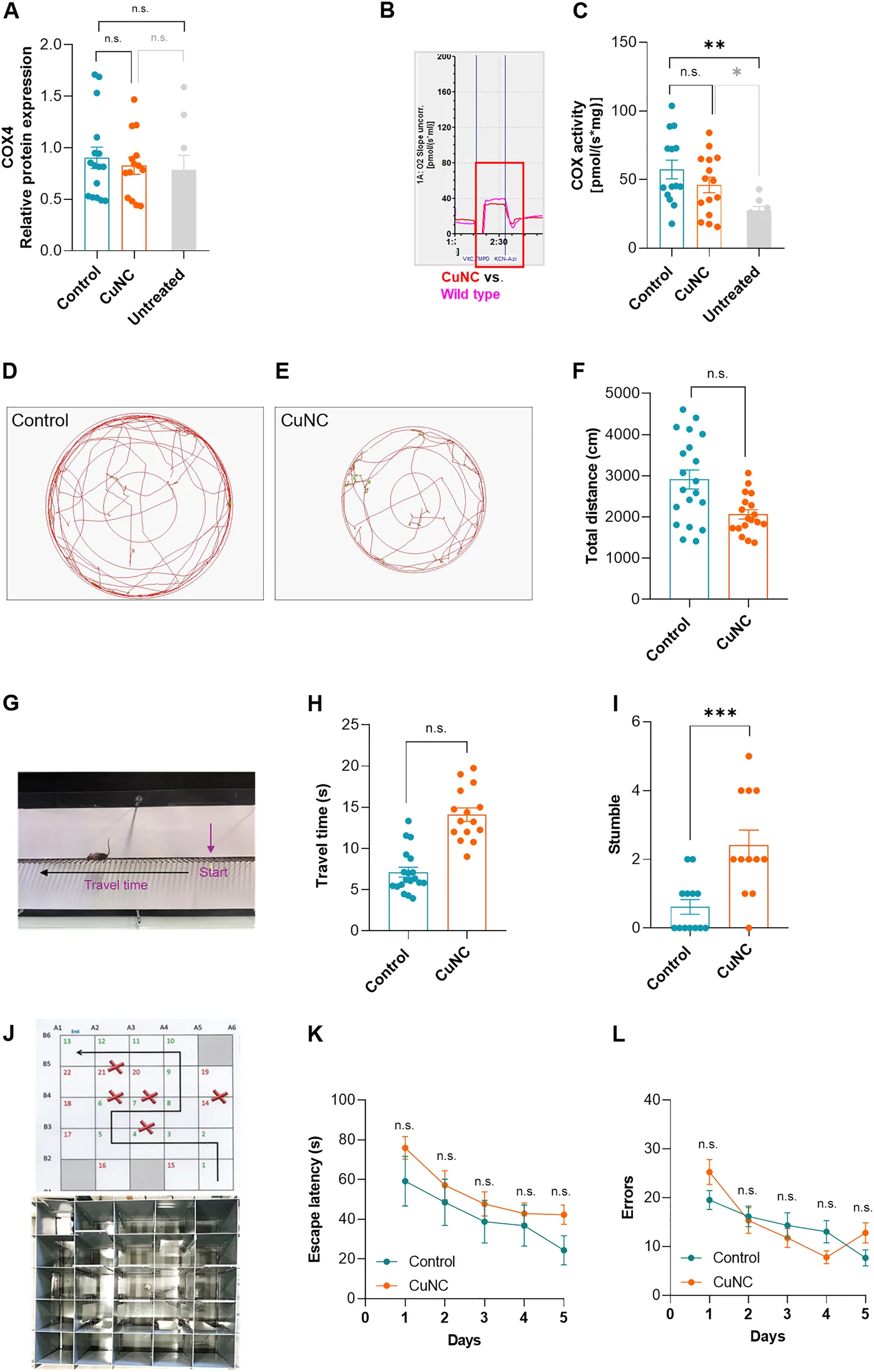
Efficacy of CuNC treatment on neurological damage. (**A**) The expression and (**B** and **C**) the activity of the cupro-dependent protein, COX, were studied by oxygraphy in the striatum and Western blot in the cortex. (**D** and **E**) Control, *n* = 14 and *n* = 16; and CuNC, *n* = 14 (PND50). The typical results of open field test obtained with wild-type mice (control; *n* = 20) or CuNCs treated Mo-blo mice (CuNC; *n* = 18) (D and E, respectively) was used to measure the explored total distance (PND27) (**F**). (**G**) Presentation of the linear walk test and (**H**) the travel time and (**I**) the number of stumbles during the crossing to determine the evolution of locomotor damage (control, *n* = 20; and CuNC, *n* = 15) (PND28). (**J**) Presentation of the aquatic maze test and cognitive impairments (memory and learning) studied by the evaluation of escape latency (**K**) and number or errors (**L**) (control, *n* = 15; and CuNC, *n* = 12; PND33 to PND38). Behavioral analyses were carried out on a minimum of control (*n* = 20) and CuNCs (*n* = 12; biologically independent samples). Statistical analyses were performed using the Kruskal-Wallis test or one-way ANOVA. n.s., not significant; *P < 0.05; **P < 0.01; ***P < 0.001.

Neurofunctional outcomes were investigated through three complementary behavioral tests. The open-field assessed spontaneous locomotor activity and general behavior (Fig. 8, D to F), the linear walking test examined motor skills and coordination (Fig. 8, G to I, and movies S7 to S9), and the aquatic maze test evaluated learning and memory process (Fig. 8, J to L) (*54*). For locomotor activity, results from the open-field test showed a decrease in total distance traveled within the test for CuNC-treated Mo-blo mice (2100 cm) compared with control mice (2900 cm), although this difference was not statistically significant, indicating that overall spontaneous locomotor activity remained preserved. For the linear walking test, CuNC-treated Mo-blo mice required an average of 14.1 ± 0.8 s to cross the test compared with 7.1 ± 0.6 s for control mice, although this difference was not statistically significant (Fig. 8H). The number of stumbles during the linear walk test was also studied and was significantly higher in CuNC-treated mice (2.4 ± 0.7) than in control mice (0.6 ± 0.2) (Fig. 8I). These results and associated movies (movies S7 to S9) demonstrate that, thanks to CuNC treatment, the muscular activity and locomotion of Mo-blo mice were comparable with those of controls, despite remaining difficulties for Mo-blo mice to perform a totally efficient locomotion. In addition, we observed that untreated Mo-blo mice exhibited clear difficulties in crossing the apparatus; however, these observations could not be robustly interpreted due to the very limited number of surviving individuals resulting from high mortality in this group (movies S8).

Neurocognitive impairments associated with hippocampal degeneration (*53*) are rarely studied in current research (*9*, *10*). In our study, they were evaluated with the water maze test (Fig. 8J). Two major parameters attested the efficient learning and memory neuronal processes of learning and memory: escape time latency and error number reduction (Fig. 8, K and L). In the context of normal neurocognitive parameters, wild-type mice (control) showed a decrease in escape latency and error numbers over the 5 consecutive days of testing. In comparison, CuNC-treated Mo-blo mice showed results not significantly different from those of the control group, suggesting a complete restoration of the altered neurocognitive parameters associated with CuNC treatment. Considering both locomotion and cognitive functions, it would be preferred to evaluate these functional outcomes in untreated Mo-blo mice. But at these ages, this group achieved higher mortality or almost no movements to perform behavioral tests, which reinforce the results of the CuNC-treated group.

In conclusion, through biochemical and neurobehavioral correlation, neurological function improvements (motricity, movement coordination, behavioral learning, and memory) of Mo-blo mice treated with CuNCs are significantly noticeable. Thus, CuNCs demonstrate their ability to cross the BBB, deliver copper into neuronal cells, and restore copper-dependent protein functions in the brain, primarily in high-energy regions such as the basal ganglia or the cerebellum, brain stem, and hippocampus, and lastly and significantly improve specific cognitive impairments in these animals.

## DISCUSSION

MD is a rare genetic disorder caused by a copper transporter ATP7A deficiency, responsible for copper depletion throughout the body, including the brain. Copper deficiency leads to impaired activities of all cuproenzymes, causing nonneurological (e.g., abnormal skin and hair, osteoporosis, and vascular damage) and neurological symptoms (e.g., epilepsy and neurodegeneration), resulting in rapid death around the age of 3 in humans. Current treatment is based on CuHis injections, which does not improve the neurological outcome in affected children (*55*). To bring copper to cuproenzymes at the nonneurological level and into the CNS, CuNCs were designed through the simple KISS chemical process, based on selected biocompatible and bio-assimilable molecules and tested in a MD mouse model after repeated subcutaneous injections. CuNCs were superior to CuHis in terms of survival. They were able to restore the activity of cuproenzymes both at the nonneurological and neurological levels. This demonstrates that copper from CuNCs reaches the cells and allows copper incorporation in apo-cuproenzymes. Our experiments highlighted a strong and significant improvement in CuNC-treated mice, although they did not completely normalize compared with control mice. Treatment at an earlier stage (antenatally) could provide even better results. These results have led to the filing of an international patent (WO2022/238656), as well as the granting of orphan drug designation by the European Medicines Agency (EU/3/22/2711) and the Food and Drug Administration (21 CFR 316.26), which will enable us to begin clinical trials in children with MD as early as possible.

## MATERIALS AND METHODS

### Materials

All reagents and solvents were of analytical grade, and they were used without further purification. Potassium phosphate monobasic and benzyl alcohol were purchased from Acros Organics (Geel, Belgium), and hydrochloric acid and sodium hydroxide solution (1.0 M) were purchase from Carl Roth (Karlsruhe, Germany). Nitric acid TraceSELECT was obtained from Fisher Scientific SAS (Illkirch, France). PTCA was obtained from BLD Pharmatech Co. (China). RNA/DNA extraction kits were purchased from Macherey-Nagel (France). The Genotyping Kit was obtained from KAPA Biosystem (The Netherlands) and the primers using from Eurogentec (Belgique). The PCR Kit was purchased from Takara (Japan). Restriction enzyme was obtained from New England Biolabs (USA). CuHis (hospital preparation, identification 77772737, batch no. T20007) was obtained from Assistance Publique-Hôpitaux de Paris. All the other reagents (e.g., copper acetate, sodium acetate, orthophosphoric acid, histidine, ascorbic acid, D_2_O, and boric acid) were purchased from Sigma-Aldrich (St. Louis, MO, USA). PBS (0.148 M) was prepared by combining 6.68 mM Na_2_HPO_4_, 1.47 mM KH_2_PO_4_, 138.00 mM NaCl, and 2.67 mM KCl. The final pH was adjusted to 7.4 with NaOH 0.1 M. Ultrapure deionized water (>18.2 MΩ·cm) was used to prepare all solutions.

### CuNC synthesis

For 2000 μl of Cu acetate (45 mg in 100 ml of water), 3000 μl of water were added. Thirty-nine milligrams of histidine was added, and the solution was agitated for 15 min. When the solution has turned blue, the pH is adjusted to 12 with NaOH 1.0 M, then 139 mg of ascorbic acid was added under argon, and the solution then turns pale yellow and was agitated at 40°C for 4 hours. All the concentrations of CuNCs are indicated as equivalent of copper metal per volume. These solutions were injected into animals.

For physicochemical characterization, the protocol was modified to reach higher concentrations. In this way, 23 mg of copper acetate was ground with 1.7 g of histidine until the obtention of a homogeneous blue powder. The obtained complex was then placed in a 50-ml reactor. Then, 3.0 g of ascorbic acid and 5 ml of water were added. The mixture was agitated for 24 hours under nitrogen until the solution became pale yellow. The initial mass of copper acetate could be adjusted from 23 to 230 mg without protocol modification depending on the following experiment. The obtained CuNCs can be used after synthesis or freeze-dried and stored at 4°C under nitrogen. When necessary, CuNCs were purified by SEC (Sephadex G-25, PD MidiTrap G-25, Cytiva) using water as eluent.

### CuNC characterization

The Malvern Zetasizer Nano-ZS system determined hydrodynamic diameters. Absorption spectra were recorded on a UV-3600 UV-visible double-beam spectrophotometer (Shimadzu, Marne La Vallée, France). Fluorescence spectra were recorded on a Fluorolog FL3-222 spectrofluorimeter (Horiba Jobin Yvon, Palaiseau, France) equipped with 450-W Xenon lamp, a thermo-stated cell compartment (25°C), and a UV-visible photomultiplier R928 (Hamamatsu, Japan). All spectra were measured in four-face quartz cuvette.

Transmission electron microscopy (TEM) images were obtained with a JEOL JEM1011 operating at 100 kV. The samples for TEM imaging were prepared by placing droplets of a dilute solution on a carbon-coated copper grid. Cryo-STEM images and EELS spectra were acquired using liquid N_2_-cooled holder in a NionHermes 200-S monochromated microscope with Cs-correction, also operated at 100 kV. The HAADF detector had a collection range of about 70 to 200 mrad. The probe size was ∼0.1 nm, with a probe convergence semiangle of 35 mrad. This setup resulted in HAADF images largely governed by *Z* contrast. EELS resolution was adjusted to about 20 meV for the collection of NCs and reference spectra. CuO and Cu2O powders and a sample of the NCs were all prepared on ultrathin holey carbon films on copper grids that were then cut into halves (NCs) and quarters (reference powders) and glued to a copper washer for mounting in the microscope. In this way, all spectra were recorded during the same session and under similar conditions. Cu metal reference spectra were acquired from the thin areas at the ends of the freshly severed grid bars. X-ray photoelectron spectroscopy (XPS) was conducted with a K-Alpha+ system (Thermo Fisher Scientific, East Grinstead, UK) equipped with a monochromatic and microfocused Al Kα x-ray source (1486.6 eV, 400-μm spot size) A pass energy of 150 eV was used for the survey scans and of 40 eV for high-resolution regions. Samples were prepared by applying several drops of the solution onto a silicon wafer.

Infrared spectra of the samples were recorded using a FTIR Bruker Equinox 55 spectrometer equipped with a Ge/KBr beam splitter and a liquid nitrogen–cooled MCT detector. To obtain the IR spectra, CuNCs were deposited on a horizontal mirror (fused silica substrate and gold coating) using an infrared microscope in reflection mode, and the spectra of the ligands were obtained using an attenuated total reflectance accessory at a spectral resolution of 4 cm^−1^.

NMR experiments were carried out to characterize CuNCs by studying histidine, ascorbic acid, and acetate ligands potentially bound to the metallic core. The relatively low sensitivity of NMR required synthesizing a concentrated solution of CuNCs at 1.6 mg/ml. This solution was then analyzed by ^1^H and ^13^C NMR before and after purification by SEC. Each of these two samples was diluted three times in D_2_O before analysis. Spectra were recorded on a Brucker 400 AVANCE at 400 MHz (^1^H NMR) and 100 MHz (^13^C NMR).

RP-HPLC with UV detection carried out ligand quantification. The purified fraction of CuNCs obtained by SEC was diluted in the HPLC mobile phase (1/10) (125 mM KH_2_PO_4_ adjusted to pH 2.5 with concentrated H_3_PO_4_) before injection. HPLC was performed on a Shimadzu Nexera LC-40 system. The diode array detector was set at 210 nm. The column (Thermo Fisher Scientific Hypersil GOLD C18, 3 μm, 250/3 mm) was heated at 40°C. The injection volume was 10 μl, and the flow rate was 0.7 ml min^−1^. Data acquisition was performed using Shimadzu LabSolution. Standard solutions (50 mM in water) of sodium acetate, histidine, and ascorbic acid were used in triplicate to obtain calibration curves from 0.30 to 1.50 mM.

The global charge of CuNCs was evaluated by capillary electrophoresis using a P/ACE MDQ CE system (Sciex) equipped with a diode array detector (ʎ = 214 nm). Measurements were performed at 25 ± 1°C using a 40-cm bare silica capillary (effective length of 34 cm) and under 10 kV (normal polarity). The background electrolyte (BGE) was a 25 mM boric acid/sodium borate buffer (pH 8.5) adjusted with 40% (m/V) 1 M NaOH. CuNCs were injected in triplicate hydrodynamically (20 mbar for 20 s). The capillary was rinsed with 1.0 M NaOH (2 min), ultrapure water (2 min), and BGE (2 min) between each injection. A neutral marker, benzyl alcohol (migration time of 2.6 ± 0.2 min, *n* = 3), was used to evaluate the electroosmotic flow. Data acquisition and processing were performed using Beckman 32 Karat software.

### Copper quantification by ICP-OES

Copper in CuNCs or in organs was quantified using the ICP-OES method. Briefly, samples (100 μl of CuNC) were first mineralized in contact with 3.5 ml of ultrapure water and 2.5 ml of 65% nitric acid. They were then digested using a Microwave Digestion System (Multiwave GO, Anton Paar) for 50 min at 210°C. The solution was recovered, and the volume was adjusted to 10.0 ml with 1% nitric acid aqueous solution. In parallel, standard solutions of copper acetate were performed in triplicate to obtain a calibration curve from 0.005 to 1.000 mg liter^−1^ in 1% nitric acid. The solutions were injected using a peristaltic pump (set at 45 rpm) in the ICP system (ICP-OES, iCAP Pro, Thermo Fisher Scientific) with argon as auxiliary gas. The nebulizing pressure was 2.1 bar, and the flow rates of the nebulizer and cooler were 0.5 and 12.5 liter min^−1^, respectively. Data acquisition was performed using Qtera software at the working wavelength of 324.754 nm.

### DFT and MoD study

The structure of the eight-atom CuNC was first fully optimized at DFT level. Its association with histidine and ascorbic acid molecules was then investigated with MoD simulations. The latter was run for 500 ns in water boxes, exploring all possible histidine residues protonation states and ascorbic acid effect on the association. A second round of MoD simulations was carried out for 1 μs for extended systems to study the self-association of CuNCs, with and without ascorbic acid. All simulations were replicated four times to ensure robust statistical analysis. Full details are available in the Supplementary Materials.

### Animals and ethical status

The experiments were carried out on Atp7a mutant mice, named Mo-blo mice or spotted mice that are described as a model of MD (*27*, *28*). Heterozygous female mice (atp7a^+/−^) (B6Ei.Cg-Atp7a<Mo-blo>/J, strain no. 002044, Jackson Laboratory) are characterized by a coat with irregular spots with alternating dark and light areas. These females have a normal metabolism, behavior, and reproductive system. They were mated to C57B6/J wild-type males (strain no. 000664, Charles River Laboratories) to allow the generation of hemizygous males (atp7a^−^) Theses mice were housed in different animal facilities according to the needs of the protocol, EOPS-type animal facilities [Central Animal Facility of the Brabois-Santé Campus (ACBS), approval no. C-54-547-22], or conventional animal facilities (Laboratory n-GERE, approval no. C-54-547-22; Animal Facility of the Hubert Curien Pluridisciplinary Institute, approval no. G-67-428-29). The animals were housed at a controlled temperature of 22° ± 1°C with a 12-hour light/dark cycle and unlimited access to food and drinking water. The experiments were carried out after obtaining a favorable opinion from the Lorraine Ethics Committee for Animal Experimentation, the Strasbourg Regional Ethics Committee for Animal Experimentation, and the Ministry of Higher Education, Research and Innovation (APAFIS nos. 32153 and 43941).

### In vivo experiments

The experimental protocol is based on implementing copper-based treatment in sick hemizygous animals from the age of 5 days till the age of 50 days and their comparison to untreated or healthy control groups. Copper dosage was calculated on the basis of the daily intake depending on age and divided by 10 to account for the absorption coefficient of copper in the intestine (*56*). IDs are presented in Table 1. The same dosages were injected in the case of CuHis-based (copper equivalent) treatments. In addition, local tolerance at the injection site was assessed by regular macroscopic clinical observation throughout the treatment period, with no visible signs of inflammation or necrosis.

**Table 1.** Injection dose of all study compounds.

Age (days)	Injection volume of the solution at 64 μg/ml (μl)	Copper supply (μg) five times/week at dose of 64 μg/ml
5 to 10	26	1.65
11 to 13	42	2.69
14 to 19	56	3.60
20 to 22	38	2.40
23 to 31	36	2.30
32 to 40	49	3.12
41 to 60	39	2.50

### Melanin analysis by HPLC-UV

Melanin analysis was performed by HPLC-UV according to the method of Ito *et al.* (*57*) with some modifications. Our analysis focuses on the production of PTCA, a component of eumelanin responsible for the black coloration of the coat. One milligram of mouse hair was weighed and placed in a tube containing H_2_O, 1 M K_2_CO_3_, and 30% H_2_O_2_ and left to stir at 37°C for 20 hours. The extraction of melanin pigments was then stopped by adding 10% Na_2_SO_3_ and 6 M H_3_PO_4_. The supernatant obtained was deposited in a black plate for analysis. A commercial standard of PTCA was prepared at a concentration of 100 μg/ml and then diluted 10-fold for further analysis. A chromatographic system (Thermo Fisher Scientific UltiMate 3000) including a binary pump, an autosampler, and a diode array detector was used. The mobile phase was applied at a flow rate of 0.6 ml/min (120 to 140 bar). It consisted of a mixture of two solutions: buffer A containing 50 mM KH_2_PO_4_, 5 mM tetrabutyl ammonium hydroxide (TBA-OH), and 4% methanol (pH 3.0); and buffer B composed of 50 mM KH_2_PO_4_, 5 mM TBA-OH, and 40% methanol (pH 3.0). Sample (10 μl) was injected in a column (Phenomenex Kinetex, 2.6 μm, C18, 100 Å, 100 mm by 4.6 mm) maintained at 40°C. The gradient of buffer B varied from 30 to 70% in 16 min. Amounts of PTCA were quantified by using a UV detector at 273 nm.

### Linear walking test

The linear walk test is based on the animals’ ability to coordinate the movements of their legs to move linearly on bars arranged horizontally between two walls. The device is a corridor delimited by two vertical walls. One of the walls is opaque and colored in white to contrast with the animal’s coat, whereas the other was transparent to allow the animal observation. The animal was placed at one end of the corridor and left to move freely. At the other end was a box containing the bedding from the housing box. Mice took the test on day 28 of age. The parameters measured were the total time taken to cross the device, and the number of times a leg (front or back) passed between the bars defined as an error. The parameters were manually recorded by the experimenter.

### Aquatic maze

Learning and memory performance were studied using an aquatic maze after the weaning period between 33 and 37 days of age. The device consists of a square tank filled with water (maintained at 25°C) with a level adapted to animal size to allow the walk and not the swim. Gray plastic walls (30 cm high) were used to delimit 25 square equal size zones (15 cm by 15 cm). Open doors in the walls allowed communication between zones to design the correct pathway and additional lateral error zones. Each mouse ran in the maze twice a day for 5 consecutive days (sessions S1 to S5 with a cutoff of 120 s). Different parameters, such as the necessary time to escape the maze (escape latency) and the number of errors (no-end zone entries or number of turn-around), attest to an efficient learning mechanism if an improvement in the parameters is observed during the successive sessions. The cutoff time was attributed if no exit was recorded (120 s). Two days before the first session, each mouse discovered the apparatus in two habituation sessions (not recorded).

### Measurement of complex IV activity by mitochondrial oxygraphy

Cellular respiration was measured with an Oxygraph-2k (Oroboros Instruments GmbH, Innsbruck, Austria). Oxygraphic analysis measured the oxygen consumption of living tissues using Clark electrodes. The electrode was calibrated before the series of manipulations and under fixed conditions (37°C with continuous agitation) for a given medium (Mir05 buffer, Oroboros Instrument GmbH). The calibration was made by adding small amounts of dithionite every 15 min in each chamber of the device to lastly stop breathing and reach the 0% oxygen level. A 0% oxygen level was achieved by the addition of a concentrated amount of dithionite, whereas 100% oxygen was obtained by opening the chamber. The mouse striatum tissues were then microdissected, weighed (∼3 mg), and immediately deposited in each oxygraph chamber containing Mir05 buffer and freshly prepared saponin (50 μg/ml). The analysis of respiration was assayed by sequentially introducing different substrates (10 mM pyruvate, 5 mM malate, 10 mM glutamate, 10 mM succinate, and 2.5 mM adenosine 5′-diphosphate) or inhibitors specific to each respiratory complex like 5 μM oligomycin A, 1.5 μM carbonyl cyanide 4-(trifluoromethoxy)phenylhydrazone, 1.5 μM rotenone, and 5 μM antimycin, and lastly ascorbic acid (11 μg/ml), *N*,*N*,*N*′,*N*′-tetramethyl-*p*-phenylenediamine (7.5 μg/ml), and 2 μM KCN to assess complex IV activity.

### Analyses of cuproenzyme expression by Western blotting: COX and LOX

The tissues were lysed by mechanical grinding using radioimmunoprecipitation assay buffer [140 mM NaCl, 10 mM Na_2_HPO_4_, 1.4 mM KH_2_PO_4_, 1% (v/v) Igepal, 0.1% (m/v) SDS, and 0.5% (m/v) sodium deoxycholate] and a cocktail of inhibitors composed of 1 mM phenylmethanesulfonyl fluoride and1 mM sodium orthovanadate as well as a cocktail of protease inhibitors 0.5% and then assayed by the BCA method. Twenty micrograms of proteins prepared in Laemmli 2X buffer containing dithiothreitol (DTT) were separated by SDS–polyacrylamide gel electrophoresis consisting of a 10% separation gel [1.5 M tris-HCl (pH 8.8), 10% (v/v) acrylamide, 10% SDS, 10% (m/v) ammonium persulfate (APS), and TEMED] and a stacking gel [0.5 M tris-HCl (pH 6.8), 0.125% (v/v) acrylamide, 0.001% (v/v) and 10% (w/v) SDS, 0.001% (v/v) and 10% (w/v) APS, and TEMED]. The proteins were transferred by semidry transfer (TransBlot Turbo, Bio-Rad) to a 0.2-μm polyvinylidene fluoride membrane. The blockage of nonspecific sites was carried out for 1 hour under slow stirring at room temperature using a 1× TBST solution containing 5% (w/v) nonfat milk. The membranes were then incubated overnight at 4°C under slow stirring with the primary antibody (COX4, Novus NB110-39115, 1:2000; LOX secreted, Novus NB100-2527, 1:500), previously diluted in the blocking solution. The secondary antibody was diluted at 1:10,000 in the blocking solution in which the membrane was incubated for 1 hour at room temperature, under gentle rocking. Regarding the reference proteins, the antibody was already coupled to horseradish peroxidase (HRP) diluted to 1:10,000 in the blocking solution and placed on the membrane for 1 hour at room temperature. The signal was quantified by chemiluminescence using a luminol/peroxide mixture in a ratio of 1:1, detectable by the iBright system (Thermo Fisher Scientific, ref. CL1500).

### Analyses of cuproenzyme expression by Simple Western: TYR and glycosylated LOX

WES (ProteinSimple, USA) enables capillary electrophoresis protein expression analysis. The various reagents, namely, DTT, Master Mix 5× fluorescent, and biotinylated ladder, were present in the supplier’s kit. The DTT was prepared by adding 40 μl of ultrapure water to obtain a 400 nM solution; the master mix was prepared by adding 20 μl of a “Sample Buffer 10×” solution provided in the kit as well as 20 μl of the previously prepared DTT solution. Last, the biotinylated ladder solution was prepared by adding 20 μl of ultrapure water. The proteins were diluted in a “dilution solution” at 0.4 μg/μl (Sample Buffer 10×/H_2_O, 1:1000). The samples were then mixed with the master mix at a 4:1 ratio and then heated to 95°C for 5 min to denature proteins. The samples were kept on ice until they were deposited on a plate. The primary antibodies used (LOX, Novus NB100-25-27, 1:250; TYR, Novus NBP2-67233, 1:250) were diluted in a dilution solution supplied in the kit at specific concentrations depending on the development. The secondary antibody (vinculin, 13901S, 1:500) was supplied in the ready-to-use kit. The peroxide/luminol mixture solution was prepared in a 1:1 ratio for deposit. The samples and reagents were placed on a specific plate for WES according to the supplier’s instructions. The plate was centrifuged for 5 min at 1000*g* and deposited in the analysis device with the migration capillaries. The analysis was then carried out using Compass for SW software.

### Colorimetric assay of plasma ceruloplasmin activity

The assay of ceruloplasmin activity (holo form) was performed on mouse plasma using a colorimetric assay kit according to the manufacturer’s recommendations (EIACPLC, Invitrogen). Briefly, 2 μl of plasma per sample were diluted in 98 μl of assay buffer, mixed with 25 μl of ceruloplasmin dyed substrate, and placed at 30°C for 60 min. Absorbance was measured using a microplate reader (Multiskan GO, Thermo Fisher Scientific) set at 560 nm. Ceruloplasmin activity was calculated using a standard curve on the basis of the activity of recombinant human ceruloplasmin and expressed as milliunits per milliliter.

### Histological analyses of wild-type and mutant mouse tissues

These analyses were performed on 5-μm tissue sections previously fixed with formalin. A control (Wilson’s disease, in which copper accumulates in organs) was added to the analysis as a positive control. The cuts were first dewaxed and then rehydrated to allow the coloring stage. The slides were then soaked in baths containing a 0.04% rhodanine solution (prepared from a 0.2% stock solution) for 24 hours at 37°C. Several reverse osmosis water rinsing baths were then carried out. The slides were then soaked in a bath of stabilized Harris hematoxylin for 5 min, rinsed with reverse osmosis water, and then bathed for 1 min in a 0.5% borax solution (prepared from a 5% stock solution). A final rinse bath was carried out before the dehydration stage. This stage began with a bath of 96° ethanol for 3 s, followed by two baths of absolute ethanol for 1 min, and ended with a bath of toluene for 5 min. Last, the sections were mounted between slides and coverslips and observed by photon microscopy.

### ALT assay in heparinized plasma from Mo-blo and control mice

Plasma ALT levels were measured using the Mouse GPT (ALT) ELISA Kit (ref. AEFI00672, Assay Genie). Plasma samples were centrifuged at 1000*g* for 15 min at 2° to 8°C within 30 min of collection. The supernatant was either used immediately or stored at −20° or −80°C until analysis. Hemolyzed or lipemic samples were excluded. Standards, properly diluted test samples (at least 1:2 with Sample Dilution Buffer), and blank controls were assigned to wells on the precoated plate, with all measurements performed duplicate. One hundred microliters of standard solutions were added to standard wells, 100 μl of Sample Dilution Buffer to blank wells, and 100 μl of diluted samples to test wells. The plate was sealed and incubated at 37°C for 90 min. After incubation, wells were washed twice with Wash Buffer without allowing them to dry. One hundred microliters of Biotin-labeled Antibody working solution was added to all wells, and the plate was incubated at 37°C for 60 min. Wells were then washed three times, allowing Wash Buffer to remain for 1 to 2 min per wash. One hundred microliters of HRP-Streptavidin Conjugate (SABC) working solution was added to each well, followed by incubation at 37°C for 30 min. After washing five times with Wash Buffer (1 to 2 min each), 90 μl of TMB substrate was added and incubated at 37°C in the dark for 10 to 20 min, until a blue color developed in the most concentrated standards. The reaction was stopped by adding 50 μl of Stop Solution to each well, turning the color yellow immediately. Absorbance was measured at 450 nm using a microplate reader. ALT concentrations were determined from the standard curve.

### β2-Microglobulin assay in heparinized plasma from Mo-blo and control mice

Plasma levels of β2-microglobulin were quantified using the Mouse B2m colorStep ELISA Kit (ref. AEFI03284). Plasma samples were centrifuged at 1000*g* for 15 min at 2° to 8°C within 30 min of collection. The supernatant was either used immediately or aliquoted and stored at −20° or −80°C until analysis, with hemolyzed or lipemic samples excluded. Standards were serially diluted to generate a standard curve, and all reagents and samples were equilibrated to room temperature (18° to 25°C) before use. Wells of the precoated plate were assigned to standards, samples, and blank controls, and all measurements were performed in duplicate. Fifty microliters of capture/detection antibody was added to each well, followed by 50 μl of standards or samples, and incubated for 60 min at 37°C. Plates were washed twice with wash buffer, and, then, 100 μl of HRP-streptavidin was added and incubated for 30 min at 37°C. Plates were washed five times with wash buffer, and 90 μl of TMB substrate was added and incubated for 10 to 20 min at 37°C in the dark. The reaction was stopped with 50 μl of stop solution, and absorbance was measured at 450 nm with correction at 570 or 630 nm if available. β2-Microglobulin concentrations were calculated from the standard curve.

### PET imaging for the study of copper biodistribution

On the day of imaging, mutant and wild-type mice received treatment that correlates with the treatment received during the 25 days of life (Table 2). All treatments were synthesized from ^64^Cu to obtain the following treatments: ^64^CuHis, ^64^Copper acetate, and ^64^CuNCs (see the Supplementary Materials for the details on production of ^64^Cu and radiochemistry). The batches were synthesized in quantities of 1 ml on the day of injection.

**Table 2.** Treatment correlation according to the experimental period. WT, wild type.

Groups	Treatment at 5 to 24 days	Treatment at 25 days
WT mice, CuNCs	No treatment	^64^CuNCs
WT mice, control	No treatment	^64^Copper acetate
Mo-blo mice, CuHis	CuHis	^64^CuHis
Mo-blo mice, CuNCs	CuNCs	^64^CuNCs

All PET/CT acquisitions were performed on an IRIS PET/CT device (Inviscan, Strasbourg, France) equipped with two octagonal rings leading to an axial coverage of 95 mm and a transverse field of view of 80 mm. The spatial resolution of the system was below 1.5 mm, with an absolute sensitivity close to 8% (NEMA standard) (*58*).

The PET acquisition protocol included two 10-min static images acquired at 4 and 24 hours after the injection of 206.8 ± 94.1 kBq/g (^18^F equivalent) of the molecule radiolabeled with ^64^Cu into the neck of the mouse. Each PET acquisition was followed by a CT acquisition (80 kV, 0.9 mA), performed for anatomical correlation. During the entire imaging procedure, the mice were kept warm and maintained under isoflurane anesthesia (2%, MINERVE company).

PET data were reconstructed using the OSEM algorithm (eight iterations, eight subsets) in a three-dimensional volume of 201 × 201 × 120 voxels. The resulting voxel size was equal to 0.42 mm in the transverse plane, while the slice thickness was equal to 0.855 mm. PET data were corrected for normalization, random coincidences, radioactive decay, and dead time during the reconstruction procedure. No attenuation or scatter corrections were applied.

A morphological volume of interest derived from the CT image was drawn to assess the mean brain activity for a semiquantitative assessment of brain uptake. All the mice were euthanized and dissected after their last imaging procedure. The organs of interest are the brain, the liver, the left kidney, the blood, and the jejunum. The brain was separated into two parts where the cortex, the hippocampus, the striatum, the brainstem, and the cerebellum were excised from the left part of the brain. Each histological specimen was weighed and placed in an automatic gamma counter (Hidex, Turku, Finland) for 1 min. The count obtained was converted to absolute activity using a calibration curve and then corrected for radioactive decay to an activity value arbitrarily taken at the time of animal sacrifice. The results are expressed as a percentage of the ID per gram of tissue (% ID/g).

### Genotyping

The mice were genotyped (at day 7) by gently removing the tail tip (≈1 mm). The genomic DNA was extracted and amplified with a KAPA Mouse Genotyping extraction kit containing an extraction buffer, a thermostable protease solution as well as a dye added to a DNA polymerase (KAPA Biosystem, The Netherlands, KK7302) used according to the supplier’s recommendations. The samples were incubated for 20 min at 75°C and then for 5 min at 95°C in a thermal cycler (C1000 Touch, Bio-Rad Laboratory, France). Amplification was achieved through addition of DNA polymerase solution and the addition of Atp7a-specific primers (forward: ACA CTT TCT TTC TTC AGC ACT GT; reverse: CCT CTG ACC TCC ACA CAT GT) also in the thermal cycler according to a specific cycle of the primer (denaturation, 3 min at 94°C; 35 cycles of denaturation, 20 s at 95°C; hybridization, 15 s at 59°C; elongation, 10 s at 72°C; ended with a final elongation step, 2 min at 72°C). The amplification step was followed by a digestion step by the Rsa I enzyme [wild allele, 137 base pairs (bp)/60pb/36 bp; and mutant allele, 197 bp/36 bp] to discriminate mutant from wild-type alleles. Samples were deposited in a 2% agarose migration gel with a DNA ladder. Electrophoresis migration was performed at 90 V for 90 min in a 1× tris-borate-EDTA buffer. The different bands were revealed under UV imaging thanks to Midori Green, a safe DNA intercalator, and an E-BOX acquisition system (CX5 TS Edge, VWR; P/N: 733-2832).

### RNA extraction and RT-qPCR analysis

Five hundred nanograms of total RNA extracted for brain tissue using Nucleospin RNA plus kit was subjected to two-step reverse transcription quantitative polymerase chain reaction (RT-qPCR) using PrimeScript RT Master Mix and SYBR Premix Ex Taq (Takara, Saint-Germain-en-Laye, France) following the manufacturer’s instructions. Primers were ordered from Eurogentec (Angers, France) (Table 3). Melting temperature was determined for each sample, and the expression of the genes of interest was normalized to housekeeping genes *GusB* and *Sub1* using Bio-Rad CFX Maestro software.

**Table 3.** Table of primers used for RT-qPCR analysis.

Gene	Primer
*Atp7a* exon 2-3	Forward: GTTTATTGCACTAGGACGATGGC
	Reverse: TAGCTTTGCAAGAGCCTCGG
*Atp7a* exon 10-11	Forward: CCGGACCATTGAACAGCAGA
	Reverse: GGAGGGTCTTTGGAGTCTGA
*GusB*	Forward: CACGGCGATG GACCCAAGAT
	Reverse: CCCATTCACCCACACAACTGC
*Sub1*	Forward: AGCAAGCGGTTCCAGAGAAG
	Reverse: TCCCGAACACTGACATATCTCA

### Statistical analysis

The Kaplan-Meier survival analysis, one-way analysis of variance (ANOVA) statistical tests, and Kruskal-Wallis test, with multiple comparisons, were performed with GraphPad Prism 8.0.1 software. All data of the in vivo studies are presented as means ± SEM, while the data of physicochemical characterization are presented as means ± SD. All the parameters studied were evaluated for statistical significance between groups according to the following criteria: *P < 0.05; **P < 0.01; ***P < 0.001; ****P < 0.0001.

## Supplementary Materials

### The PDF file includes:

Figs. S1 to S8 Supplementary Text Tables S1 to S3 Legends for movies S1 to S9 References
