# Supplementary material for: Restoring brain functions in the Mo-blo mouse model of Menkes disease with copper nanoclusters
Source: Sci Adv. 2026 Sep 30;12(40):eaeg6972. doi: 10.1126/sciadv.aeg6972 (PMC13626065; doi:10.1126/sciadv.aeg6972)
Supplement: Supplementary file 1 [file sciadv.aeg6972_sm.pdf]

Science Advances
AAAS

Supplementary Materials for

Restoring brain functions in the Mo-blo mouse model of Menkes disease with copper nanoclusters

Audrey Malardé *et al.*

Corresponding author: Ariane Boudier, ariane.boudier@univ-lorraine.fr

Sci. Adv. **12**, eaeg6972 (2026)
DOI: 10.1126/sciadv.aeg6972

The PDF file includes:

Figs. S1 to S8
Supplementary Text
Tables S1 to S3
Legends for movies S1 to S9
References

Other Supplementary Material for this manuscript includes the following:

Movies S1 to S9

I. Supplementary figures

A. NMR analyses

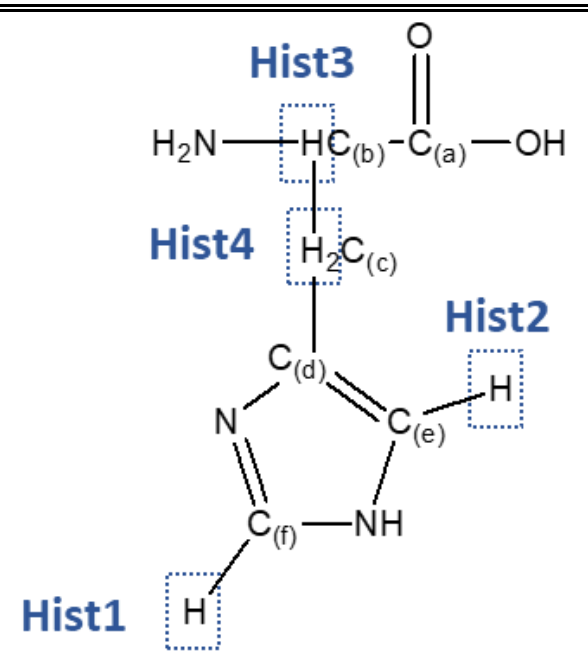

Histidine (Hist)

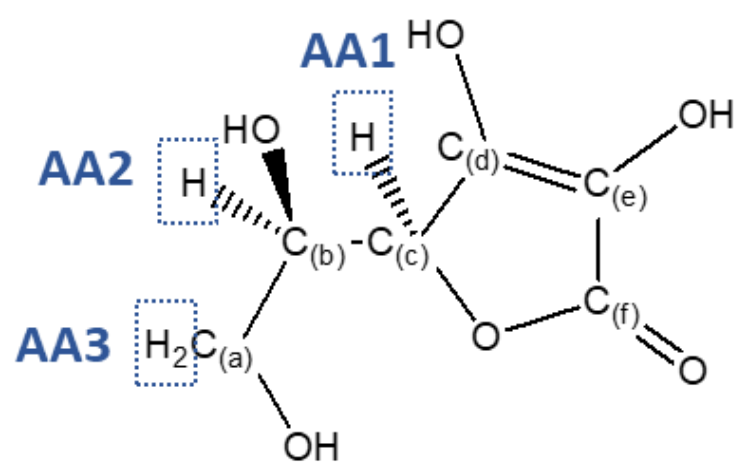

Ascorbic acid (AA)

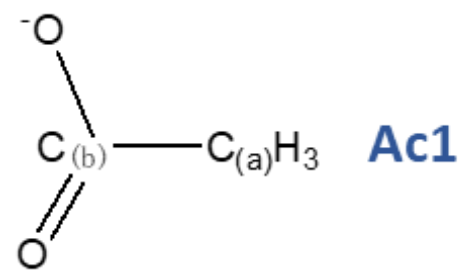

Acetate (Ac)

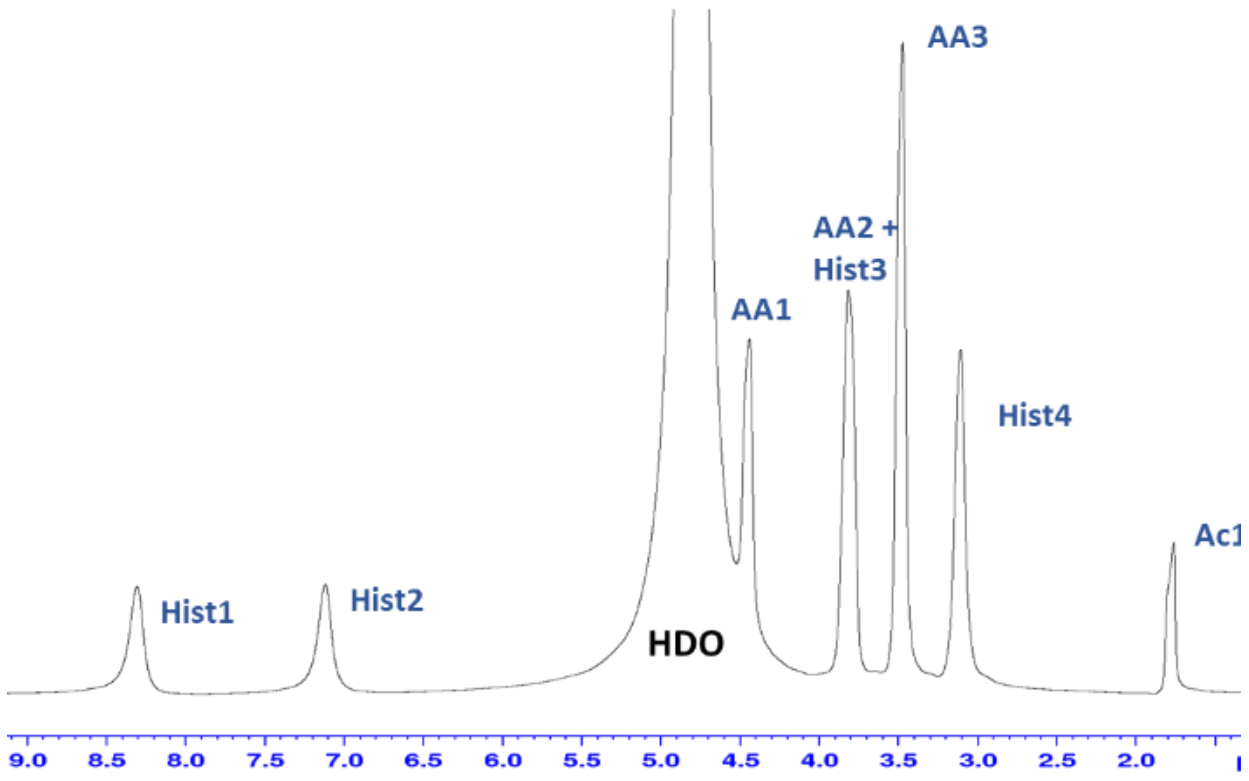

^{1}H NMR spectrum (D_2O, 400 MHz). Unpurified sample

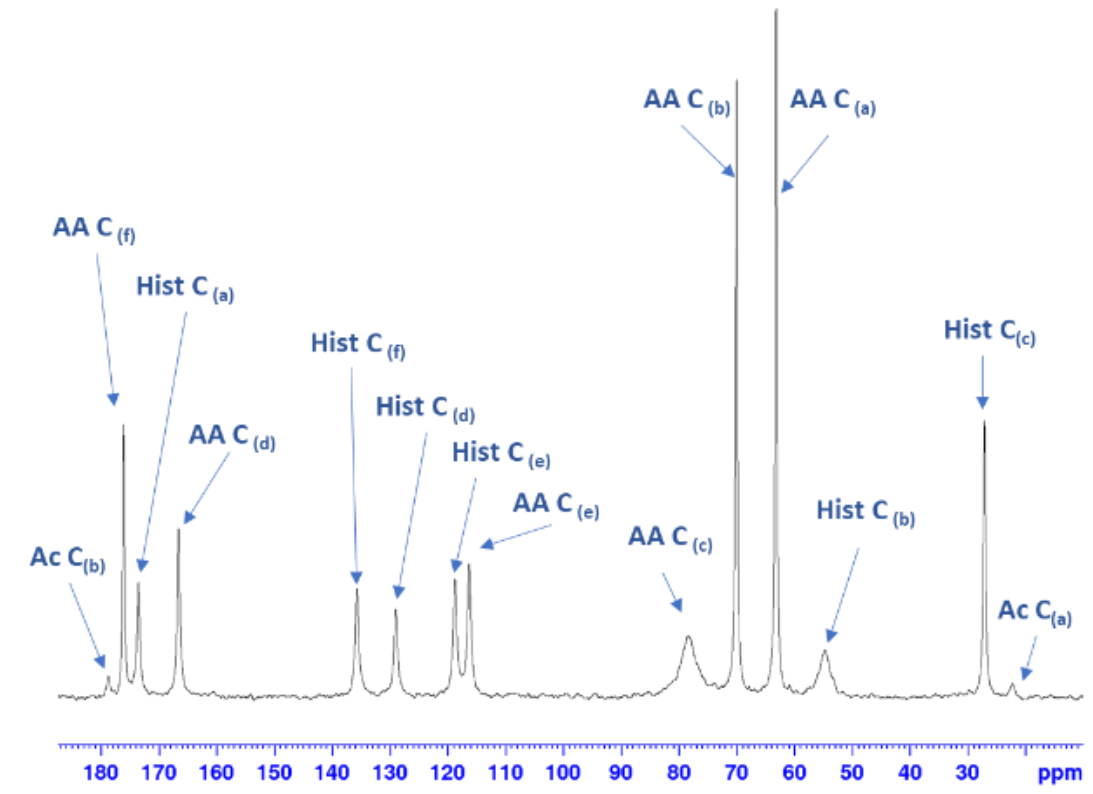

^{13}C NMR spectrum (D_2O, 100 MHz). Unpurified sample

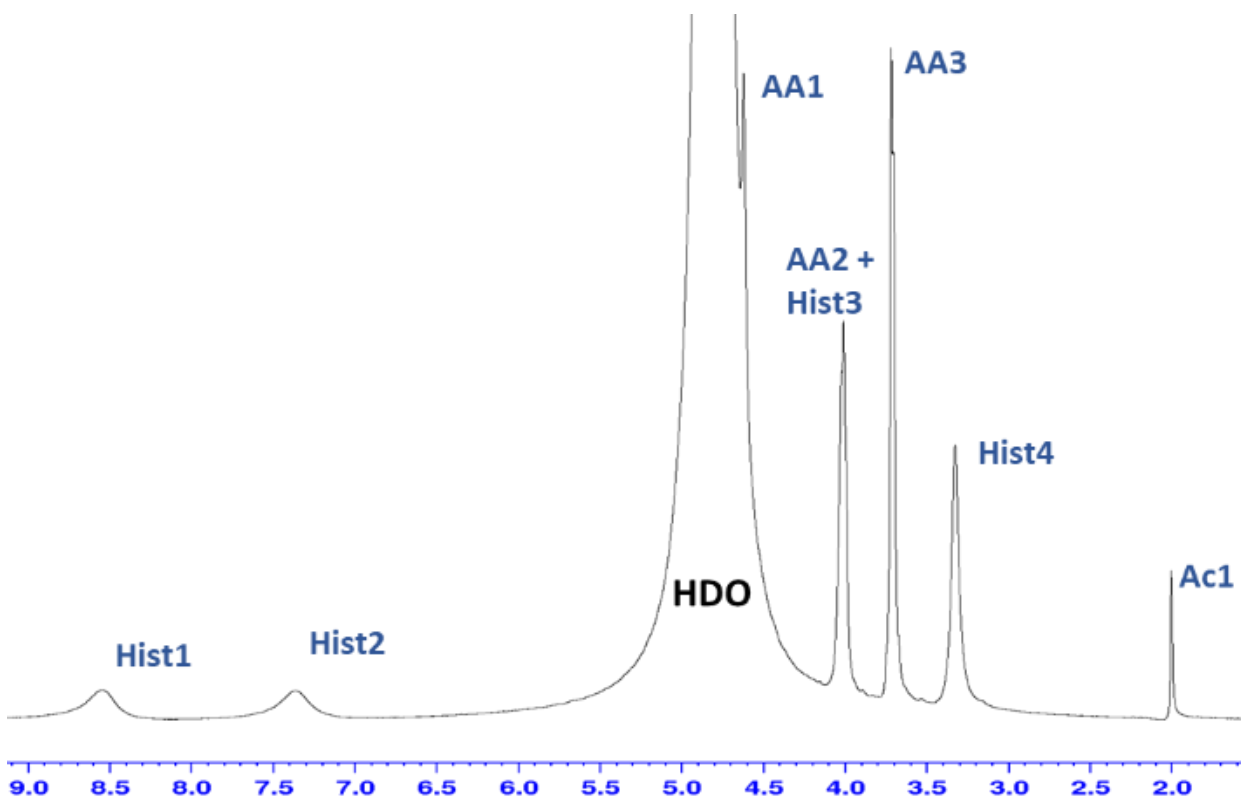

^{1}H NMR spectrum (D_2O, 400 MHz). Purified sample

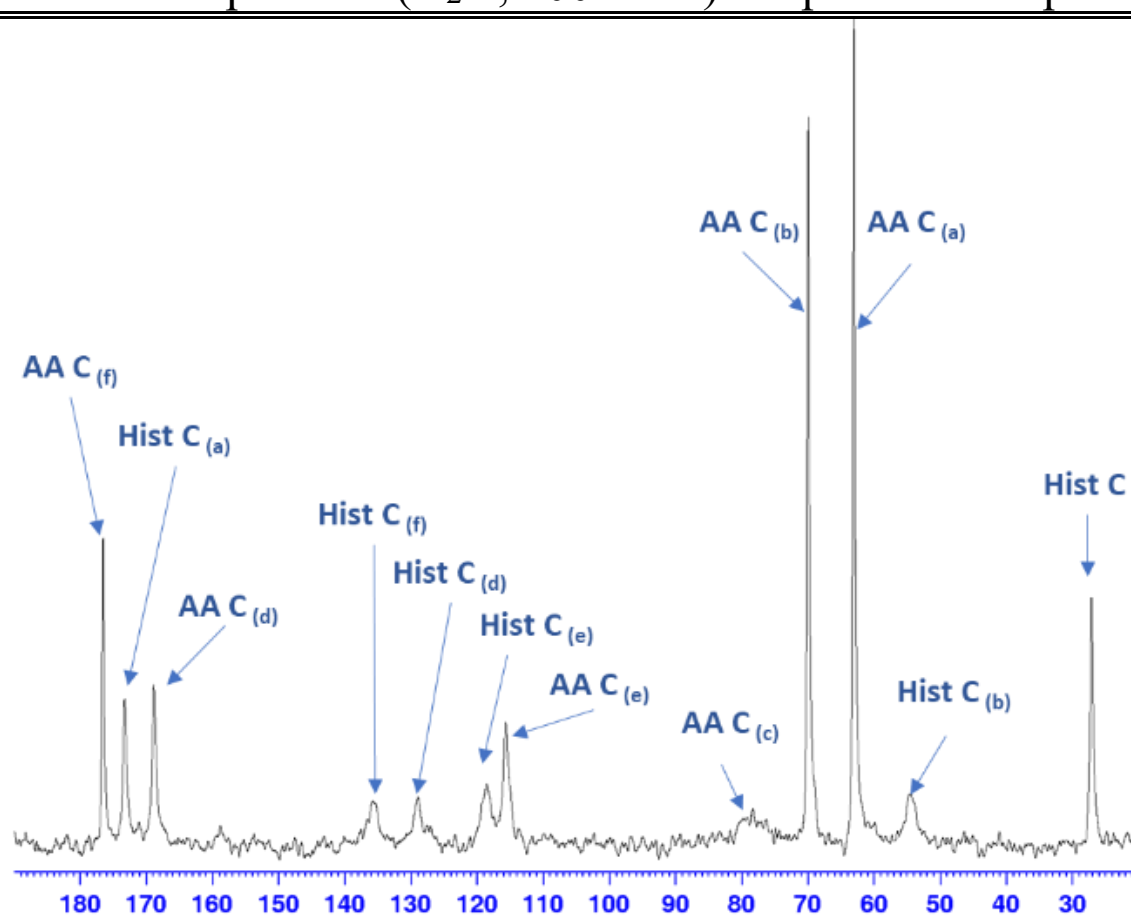

^{13}C NMR spectrum (D_2O, 100 MHz). Purified sample

	Ligand (from literature)		Copper NC ^{1}H NMR (D_2O, 400MHz)			
			Purified		Unpurified	
	A- d [ppm]	AH d [ppm]	d [ppm]	Rel. Int.	d [ppm]	Rel. Int.
Hist1	7.74(60)	8.67(60)	8.54	1.00	8.31	1.00
Hist2	7.04(60)	7.39(60)	7.36	1.01	7.12	0.96
AA1	4.56(61)	4.97(61)	4.62	0.70	4.44	1.11
Hist3 AA2	3.98(62) 4.03(61)	4.08(61)	4.02	2.57	3.81	2.26

	Ligand (from literature)		Copper NC ^{13}C NMR (D_2O, 100 MHz)	
	A- d [ppm]	AH d [ppm]	Purified d [ppm]	Unpurified d [ppm]
Ac $C_{(b)}$	184.3(63)	177.2(64)	-	178.8
AA $C_{(f)}$	180.3(65)	174.0(66)	176.6	176.2
Hist $C_{(a)}$	172.1(67, 69)	174.8(67, 68)	173.3	173.6
AA $C_{(d)}$	178.5(65)	156.0(66)	168.9	166.6

AA3	3.76(61)	3.75(61)	3.72	3.13	3.47	2.81
Hist4	3.18(62)		3.33	2.15	3.11	1.84
Ac1	1.92(60)	2.08(60)	2.00	0.36	1.76	0.63

Hist $C_{(f)}$	135.0(67, 68)	137.2(67, 68)	135.8	135.8
Hist $C_{(d)}$	127.7(67, 68)	133.1(67, 68)	128.9	129.0
Hist $C_{(e)}$	118.9(67, 68)	117.7(67, 68)	118.6	118.8
AA $C_{(e)}$	115.8(65)	118.7(66)	115.7	116.3
AA $C_{(c)}$	81.1(*65*)	77.0(66)	78.3	78.4
AA $C_{(b)}$	72.3(*65*)	69.8(66)	69.9	70.0
AA $C_{(a)}$	65.3(*65*)	62.9(66)	63.0	63.2
Hist $C_{(b)}$	53.6(67, 68)	55.7(67, 68)	54.4	54.7
Hist $C_{(c)}$	26.2(67, 68)	29.0(67, 68)	27.1	27.1
Ac $C_{(a)}$	26.0(63)	21.0(64)	-	22.3

Figure S1: Analyses of CuNCs by ^{1}H and ^{13}C NMR.

B. Study of the auto-association of CuNCs by DLS

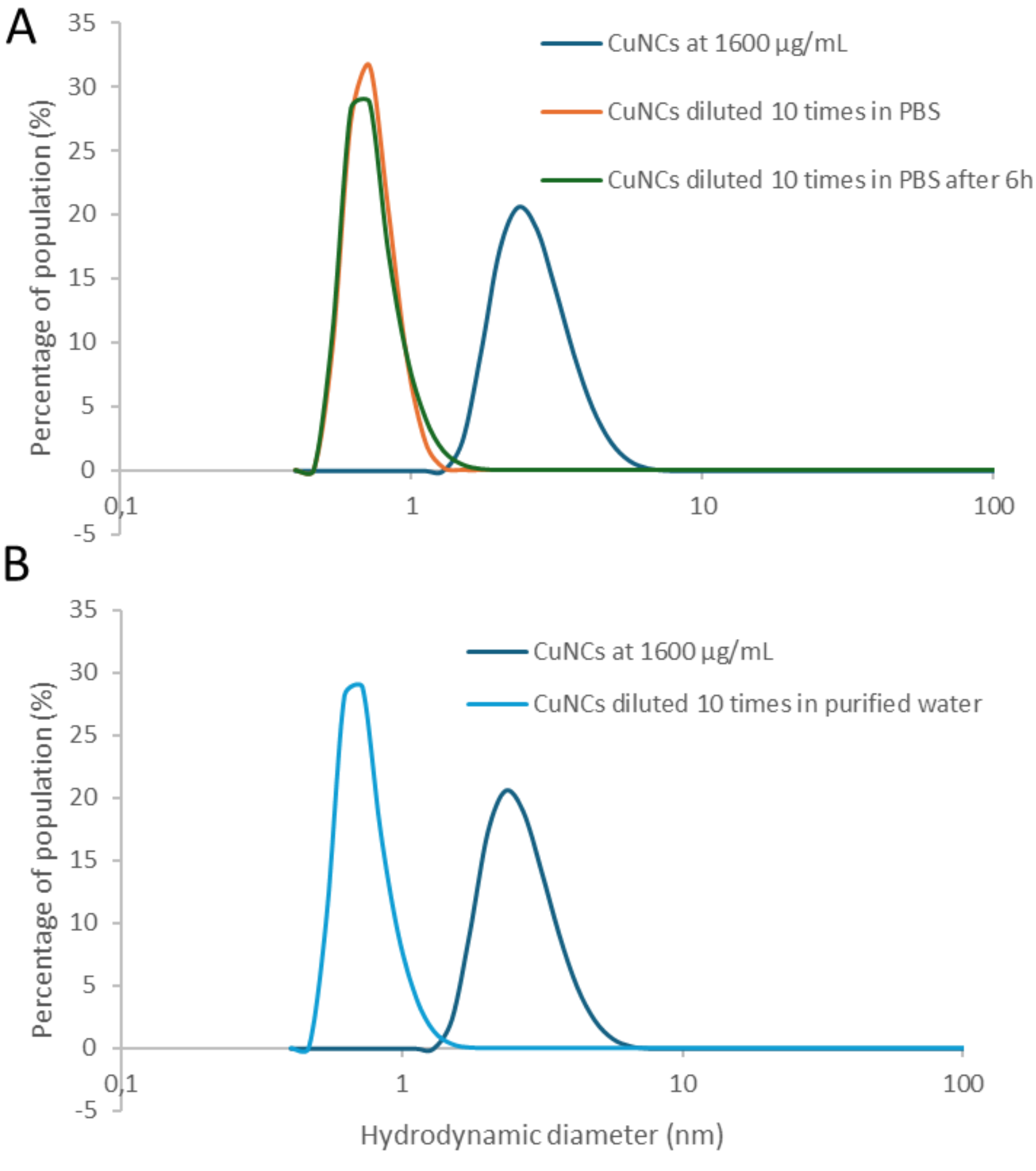

Figure S2: Analyses in DLS of CuNCs initially at 1600 µg/mL of copper and diluted in PBS or purified water (final concentration at 160 µg/mL).

C. <u>Long-term stability studied by DLS.</u>

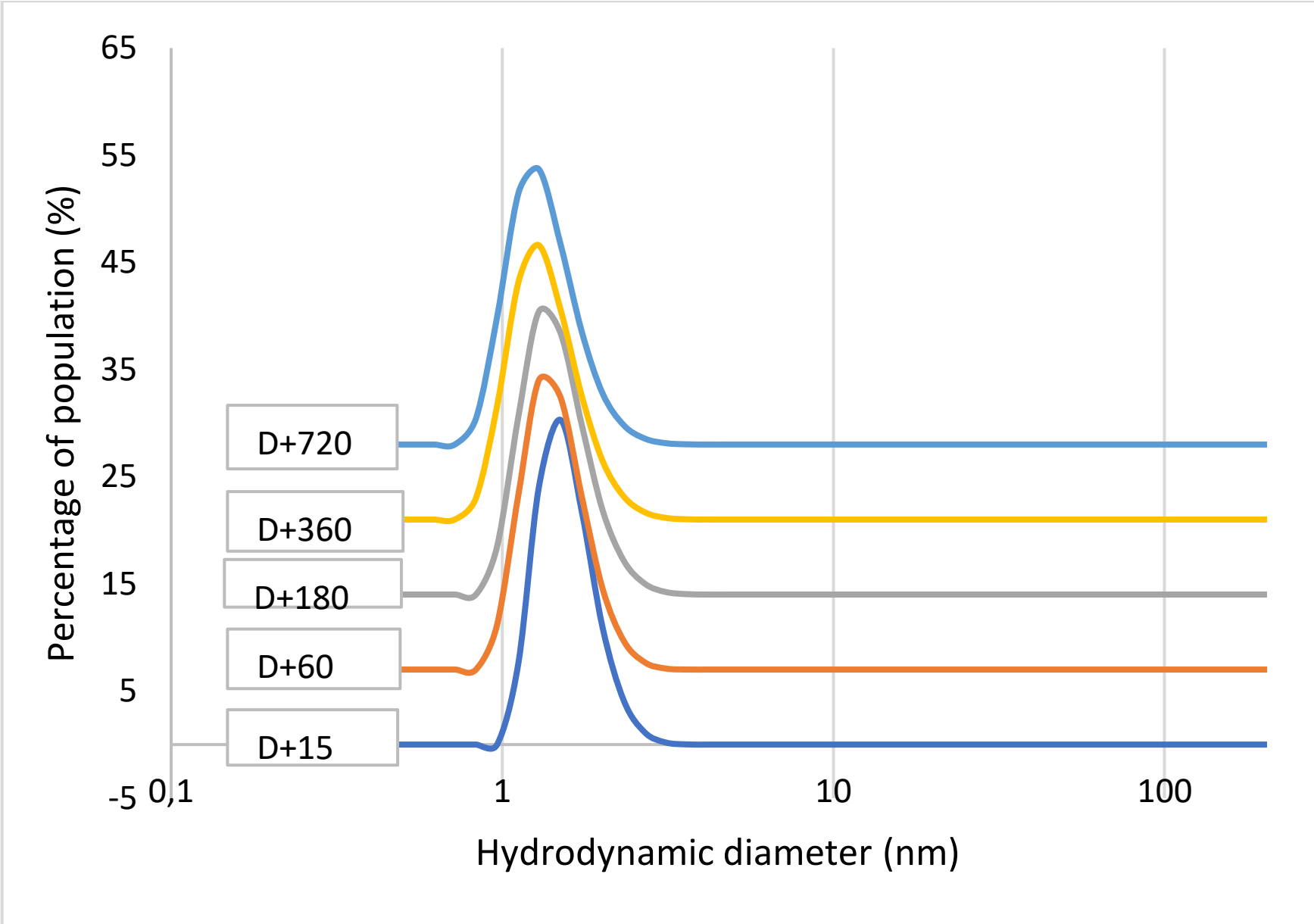

Figure S3: Long-term stability stored freeze dried at 4°C with nitrogen and resuspended in purified water at days 15, 60, 180, 360 and 720 after synthesis.

II. Supplementary data of simulation

A. Computational details

The structure of the 8-atom CuNCs (Cu_8) was fully optimized at DFT level using the release C.01 of the Gaussian software(69). The computations were performed with the PBE0 exchange-correlation density-functional approximation(70) and def2-SVP double-zeta basis set(71). The resulting minimum energy structure was then used in the molecular dynamics' (MoD) simulations reported below.

MoD simulations were used to study the dynamic association of histidinate and ascorbate on copper nanoclusters. At the experimental pH, histidine residues were considered in their zwitterionic form with two different states: one where the histidine side chain was neutral and the other where it was protonated and thus carried a positive charge. While ascorbic acid was found in the form of ascorbate and thus carried a negative charge. All simulations were performed using version 24 of the Amber software(72). To preserve the structure of the 8-atom CuNCs and maintain the electronic structure derived from quantum calculations, small harmonic constraints were applied with a force constant of 1 kcal.$Å^{-2}$.mol^{-1}. The INTERFACE(73) force field was used to model the Cu_8NC, while the histidine residues were treated with the Horn's force field(74), designed for the study of isolated amino acids. Finally, the GAFF2(75) force field was used to model ascorbate, with partial atomic charges derived from a semi-empirical QM calculation with bond correction(76). All systems were embedded in TIP3P(77) water boxes with periodic boundary conditions.

Two types of studies were performed: the first one focused on the specific interaction of a Cu_8NC with different types of residues, and the second one examined the self-assembly of multiple Cu_8NC units in an environment close approximates to experimental conditions. All simulation systems were created using Packmol software (78), which randomly places residues around Cu_8NC units. Five hundred ns MoD was used for the smaller systems, *i.e.*, those containing only one CuNCs, while 1.1 µs MoD was used for the larger systems, which investigated the self-assembly of multiple Cu_8NC units. All simulations were reproduced with 4 different starting structures to provide a robust statistical view of the results. The CPPTRAJ software (79) and Python scripts were used to analyse the trajectories, while the VMD software(80) was used to visualize the MoD results and generate figures. All simulation

systems are listed in **Table S1**. Root Mean Square Deviation (RMSD) curves and classical energy and density observations were performed for each simulation to ensure proper MoD equilibration and systems stabilization. All structural analyses were performed on the last 100 ns of the trajectories.

Table S1: Summary of the names of the systems studied with their respective volume (in nm^3), number of NC, neutral histidine residues (His), histidine protonated on its imidazole ring (His+) and ascorbate (Asc).

System name	Volume	Cu8	His	His+	Asc
**CuHis**	64	1	20	0	0
**CuHis+**	64	1	0	20	0
**CuHH**	64	1	10	10	0
**CuHHA**	64	1	10	10	10
**sCuHH**	1728	27	270	270	0
**sCuHHA**	1728	27	270	270	270

To evaluate the ability of Cu_8NCHis to cross the blood–brain barrier (BBB), MoD simulations were performed in explicit solvent under periodic boundary conditions. Following the approach proposed by Wang et al.(43), we constructed a representative model of the apical membrane of the BBB using CHARMM-GUI. The membrane was composed of 16 POPC (1-palmitoyl-2-oleoyl-sn-glycero-3-phosphocholine), 32 SLPC (1-stearoyl-2-linoleoyl-sn-glycero-3-phosphocholine), 32 SAPC (1-Stearoyl-2-Arachidonoyl-sn-glycero-3-Phosphocholine), 24 SOPE (1-Stearoyl-2-Oleoyl-sn-glycero-3-Phosphoethanolamine), 56 SAPE (1-Stearoyl-2-Arachidonoyl-sn-glycero-3-Phosphoethanolamine), 32 SAPS (Stearoyl-arachidonoyl-phosphatidylserine), 8 SAPI (1-Stearoyl-2-Arachidonoyl-sn-glycero-3-Phospho-1'-myo-inositol), 72 OSM (N-oleoyl-d-erythro-sphingosylphosphorylcholine), and 112 cholesterol molecules. The AMBER Lipid11(82) and Lipid21(83) force fields were employed to describe the membrane, together with the TIP3P water model (77). MD simulations were carried out for 1 µs trajectories and reproduced over five independent replicas.

B. Results and discussions

1. *DFT computations*

Figure S4 shows how the HOMO-LUMO gap changes with the size of the CuNC. As the number of copper atoms increases, the HOMO-LUMO gap generally decreases, indicating that the nanoclusters exhibit more metallic behaviour. However, local maxima observed at clusters containing 8 and 14 copper atoms suggest enhanced stability at these specific sizes.

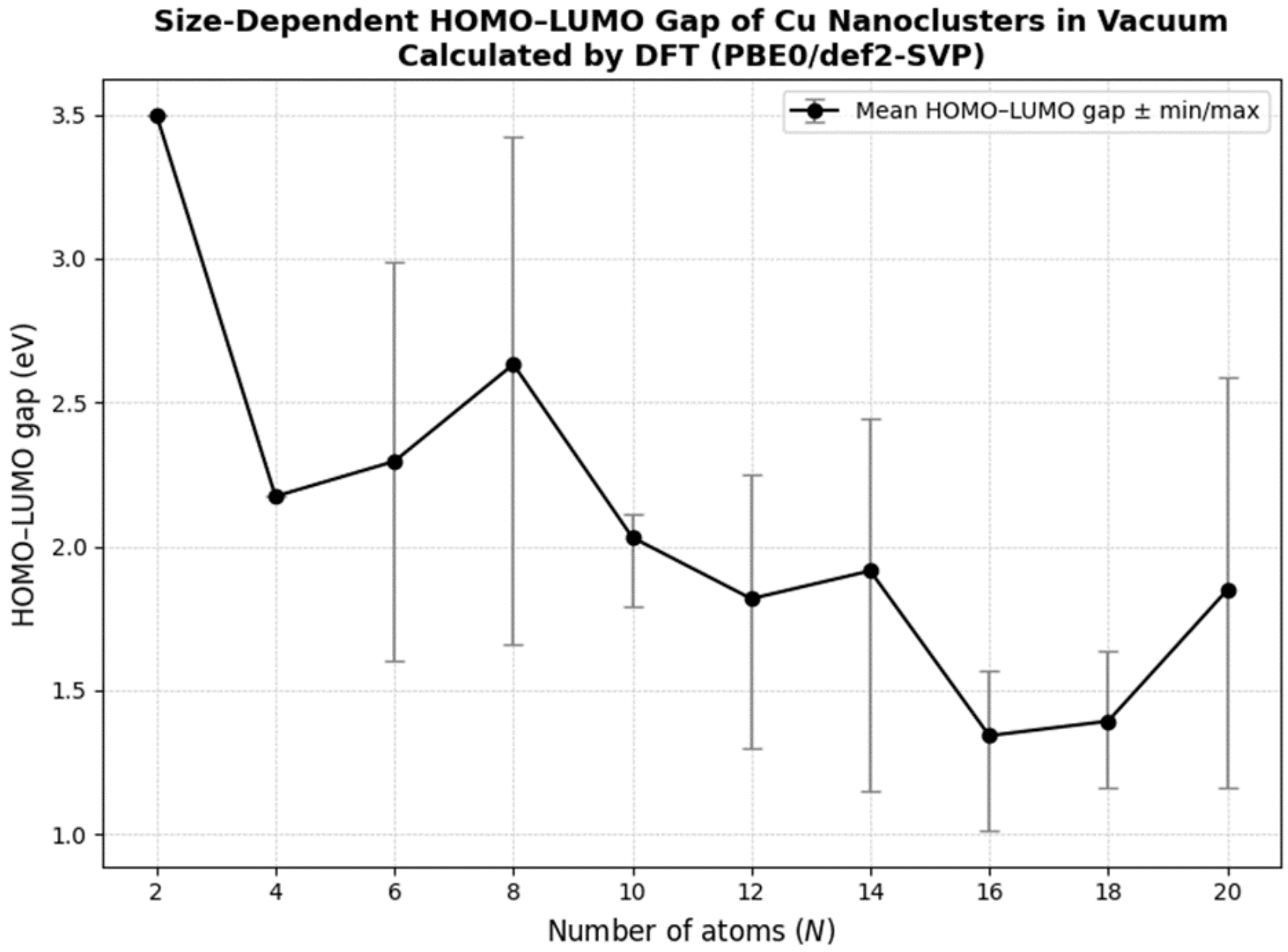

Figure S4: DFT calculation of the energy gap, in eV, between the lowest unoccupied molecular orbital (LUMO) and the highest occupied molecular orbital (HOMO) according to the CuNC number of atoms.

2. *MoD computations*

Molecular dynamics simulations were performed to investigate the association of Cu_8NC with different species (**Movie 1, 2 and 3**). These simulations were made over extended trajectories with durations of 500 ns for systems containing a single Cu_8NC, and 1.1 µs for systems containing multiple NCs. The full set of investigated systems is shown in **Table S2**. Calculations were performed using four different starting points to ensure a statistically reliable overview of the results. For all systems, stability was confirmed at the energy and density levels, with RMSD variations monitored over time. An example of this analysis is shown in **Figure S5 A**. All results converged on system stability, allowing the focus to be on structural analyses. These analyses were divided into two parts: the study of the association of different species with a single CuNCs and the study of their self-assembly.

3. *Study of association:*

For the CuHis, CuHis+, CuHH, and CuHHA systems, the RMSD curves diverged rapidly from the initial structure, which is consistent with the intrinsic plasticity of individual residues. The curves for the four replicas of the CuHHA system are shown in **Figure S5**.

Even if the RMSD reached a plateau, curves were highly blurred indicating large RMSD fluctuations consistent with a lack of structural convergence. Visualization of MoD trajectories revealed that for all considered systems, a unique structure was not attained, but instead a significant structural exchange occurred between the histidine and, where applicable, ascorbate residues around the NC.

The visualization of trajectories confirmed the flexibility of our systems, as identified by the RMSD. Consequently, the histidine residues, regardless of their protonation state, along with ascorbate where applicable, approach the NC during the first 10 ns and engage in various interactions with it. A single structure was not observed; rather, different types of associations were formed, notably involving stacking interactions between the aromatic rings of histidine residues and CuNCs. Numerous van der Waals interactions were also observed between the ligands and the NC. As a result, the NC was almost continuously covered by the ligands, thereby demonstrating CuNCs strong affinity for histidine residue and ascorbate. Finally, stabilizing interactions, particularly of an electrostatic nature, were established among the ligands themselves, involving the positively charged ammonium groups of the peptide backbone (N-terminal) and the protonated imidazole rings of His+ residues with the negatively charged regions of ascorbate and the carboxylate groups of the peptide backbone (C-terminal). **Figure S5B** illustrates these typical interactions.

It is noteworthy that regardless of the considered system, the NC is almost permanently covered by ligands. Furthermore, in the CuHH and CuHHA systems, where multiple ionization states of histidine residues are present, it was observed that the CuNCs associated with both types of histidine residues without selecting a particular protonation state. This indicates that the nanocluster is not selective regarding the surrounding ligands but rather finds an opportunity to interact with each of them. In addition, it should be emphasized that no definitive structure was observed in any of the considered systems. Instead, the process was dynamic, with ligands continuously exchanging around the

nanocluster while maintaining a significant number of interactions. The dynamic nature of the association between ligands and CuNCs requires a statistical analysis of the interaction. The number and type of residues associated with the NC throughout the dynamic process have been recorded. The visualization of the number of residues along the trajectory analysis has also been observed, revealing that the number of residues, although fluctuating, remained consistent throughout the dynamics, as illustrated in **Figure S5C**. The stability of the systems enabled the averaging of all values and the determination of their standard deviations. These values, which represent the stoichiometry of the association with the NC, are summarized in **Table S2**.

Experimentally (from HPLC-UV), there were 14.5 histidine residues around the CuNCs. From the simulation data compiled in **Table S2**, a similar order of magnitude was observed, albeit with a slightly lower number of histidine residues than experimentally found. Considering the experimental pH, it was necessary to study the association with a mixture of neutral and protonated histidine. Surprisingly, this consideration did not lead to a reduction in the number of ligands around the NC in the CuHH system. When ascorbate was present (CuHHA system), it is observed that ascorbate interacts with the NC and is, therefore, not merely a spectator ion. Furthermore, ascorbate did not significantly reduce the number of histidine residues around the NC, suggesting that ascorbate plays a role in stabilizing histidine around the NC, further highlighting its essential role in the synthesis of our assemblies.

Table S2 : Stoichiometry of histidine and ascorbate residues determined from MoD simulations

**Residue/System name**	**CuHis**	**CuHis+**	**CuHH**	**CuHHA**
**Histidine**	9.6 ± 1.7	6.8 ± 1.2	9.1 ± 1.6	8.7 ± 1.2
**Ascorbate**	0	0	0	4.0 ± 1.1

Bolstered by the consistency of the modelling results, it was decided to delve further into this approach and explore whether it would be possible to observe an association involving multiple CuNCs, drawing inspiration from our recent work(41). Consequently, two significantly larger simulations were undertaken, each encompassing 27 CuNCs with the same proportion of histidine residues (in both states

previously considered). The second simulation was distinguished from the first simulation by the addition of ascorbate to the system. The simulation process is exemplified in **Figure S5D and E**, where the positioning of CuNCs was shown at time 0 of the MoD and after 1 µs. In all cases, an association of the NCs was observed, though it was not complete (**Movie 4, 5 and 6**). This observation remains consistent with the particularly plastic nature of the association with the NCs that was observed with a single unit.

When visualizing the trajectories of each system, it was observed that, without the addition of ascorbate, the aggregates appeared to be of smaller dimensions. To quantify this, the number of CuNCs within the aggregates was counted and averaged, with the results presented in **Table S3**. This table clearly shows that the percentage of NCs remains non-associated. Once again, this is consistent with the plastic nature of the associations observed with CuNCs. The addition of ascorbate has a significant effect on the size of the observed NC assemblies, leading to aggregations ranging from 8 to 10 NC units. Ascorbate, by its anionic nature, establishes long-range electrostatic interactions, enabling salt bridges with the cationic parts of histidine, particularly those with a protonated side chain. It also screens positive charges, thereby facilitating the approach of different units.

Table S3 : Percentage of association for the systems with and without ascorbate

	**% Without Asc**	**% With Asc**
**1 NC**	30.6	26.3
**2 to 4 NC**	49.3	28.0
**5 to 7 NC**	20.1	23.7
**8 to 10 NC**	0.0	22.0

It is interesting to note that aggregates with fewer than 4 to 6 units are generally spherical in shape. However, once this number is exceeded, more massive aggregates (as encountered in simulations with ascorbate), and clearly ellipsoidal shapes are observed. The dimensions of these ellipses closely resemble those observed experimentally. For instance, **Figure S5F** shows an 8-unit aggregate next to

an ellipse with the following dimensions a=b=0.35, c=1.0 nm, demonstrating the excellent reproduction of experimental data.

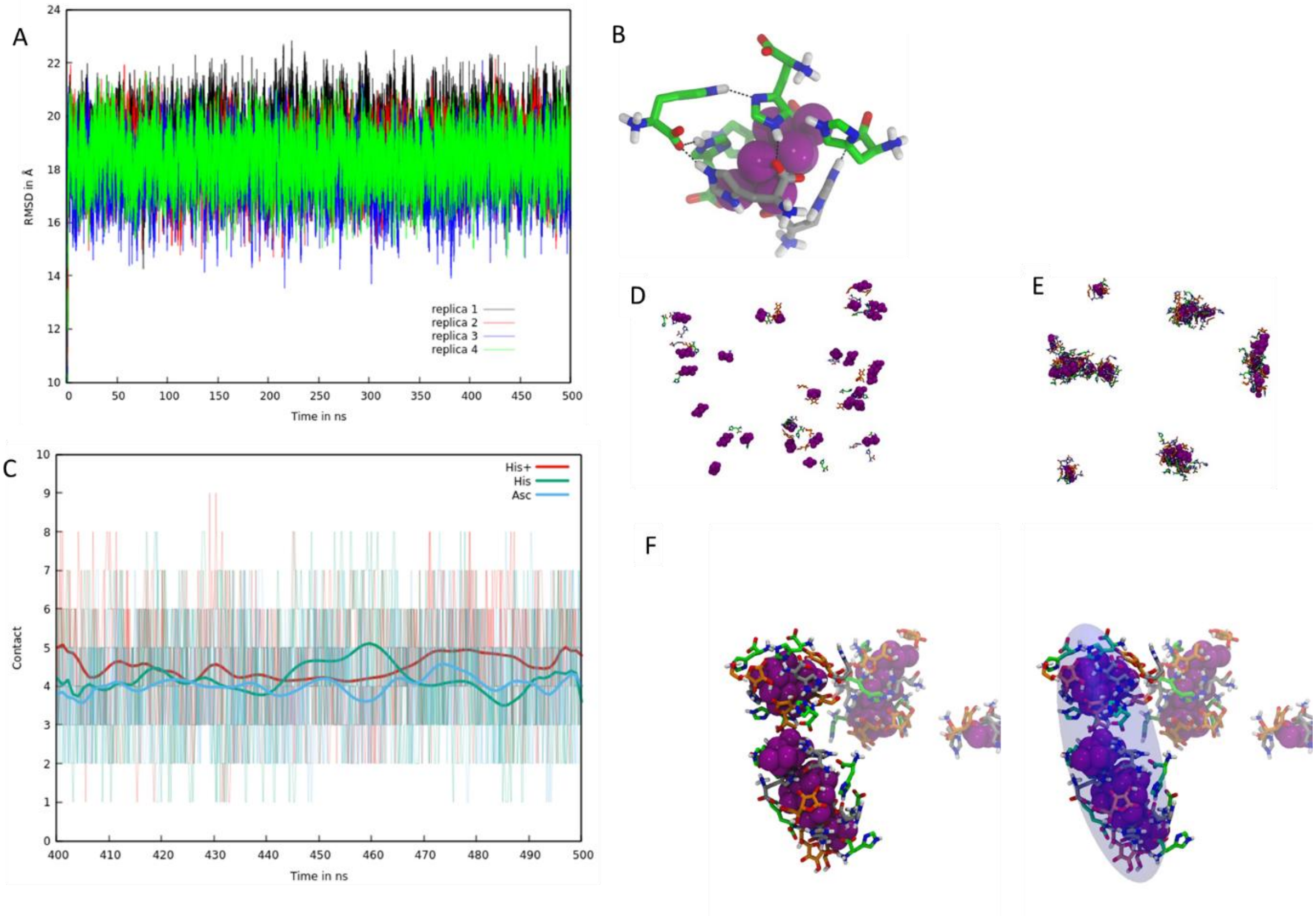

Figure S5: (**A**) RMSD *vs* trajectory time curves for the four replicas of the CuHHA system; (**B**) Typical visualization of the association of NC with histidine residues, Cu_8 NC is displayed with vdW spheres in purple while histidine residues are represented by sticks. Protonated and neutral histidine residues have their carbons in grey and green, respectively. Only polar hydrogens are displayed and hydrogen bonds are represented by dashed lines; (**C**) Example of number of residues which interact with NC along the last 100ns of MoD trajectory (here, one replica of the CuHHA system); Example of aggregation process: (**D**) and (**E**) represent the NC structures (purple) surrounded by histidine and ascorbate residues at the beginning and the end of MoD; (**F**) Example of ellipsoidal shapes found in the aggregates for the MoD simulations with ascorbate. Same representation than in B with the carbons of ascorbate depicted in orange. An ellipse of dimension of diameters (a=b=0.7, c=2.0 nm) is shown in blue.

4. *Study of CuNC (CuHHA) interaction with BBB membrane*

Following the construction of the BBB membrane model, the system was equilibrated in explicit solvent. Visual inspection revealed that the membrane remained stable throughout the equilibration phase,

displaying only minor structural fluctuations. To further assess the stability of this model, we report in **Figure S6** the area per lipid as a function of simulation time, a classical metric for evaluating membrane stability (41). As shown in this figure, during the first ~5 ns of simulation, the membrane undergoes a slight compression before stabilizing around an average value of 52.75 Å². This behaviour indicates a particularly stable membrane surface, onto which a CuHHA structure was subsequently positioned at a distance of approximately 20 Å above the bilayer surface.

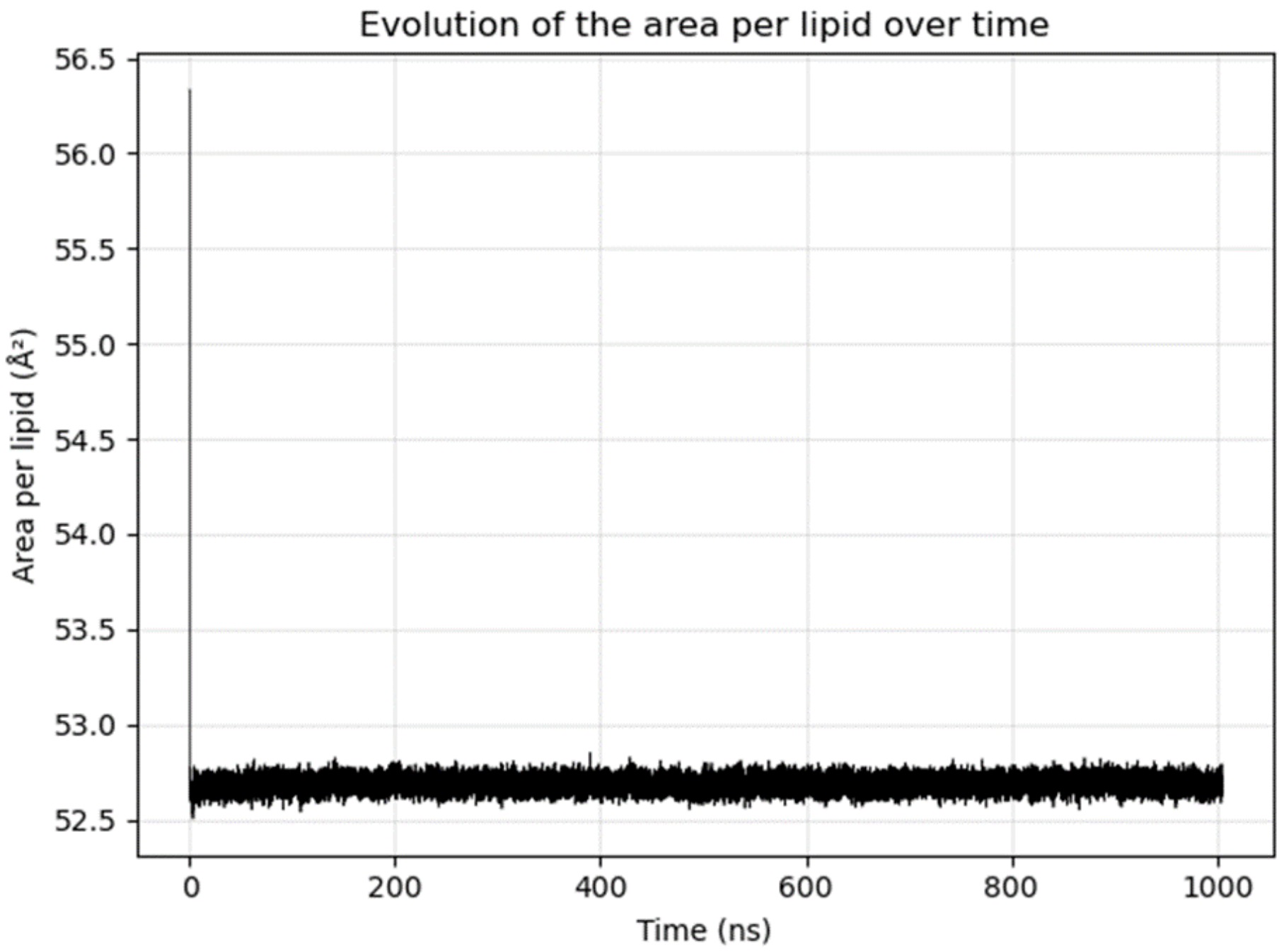

Figure S6: Area per lipid of the BBB membrane with solvent and without CuNC.

Five independent replicas were produced, each with a distinct CuNC structure. Nevertheless, the same phenomenon was consistently observed. During the first hundred nanoseconds, the CuNC remained intact while progressively approaching the membrane surface. Subsequently, the histidine and ascorbic acid ligands dissociated from it as they established stabilizing interactions with the polar and charged headgroups of the phospholipids. As the CuNC gradually lost these coordinating ligands, it penetrated the membrane, taking advantage of the hydrophobic environment provided by the alkyl chains of the lipid bilayer to remain embedded within the BBB membrane. This process occurred within less than 300 ns and is illustrated in a supplementary movie.

The interaction of CuNC with the lipid membrane does not induce any noticeable perturbation, as evidenced by the ^{31}P density profiles shown in **Figure S7**.

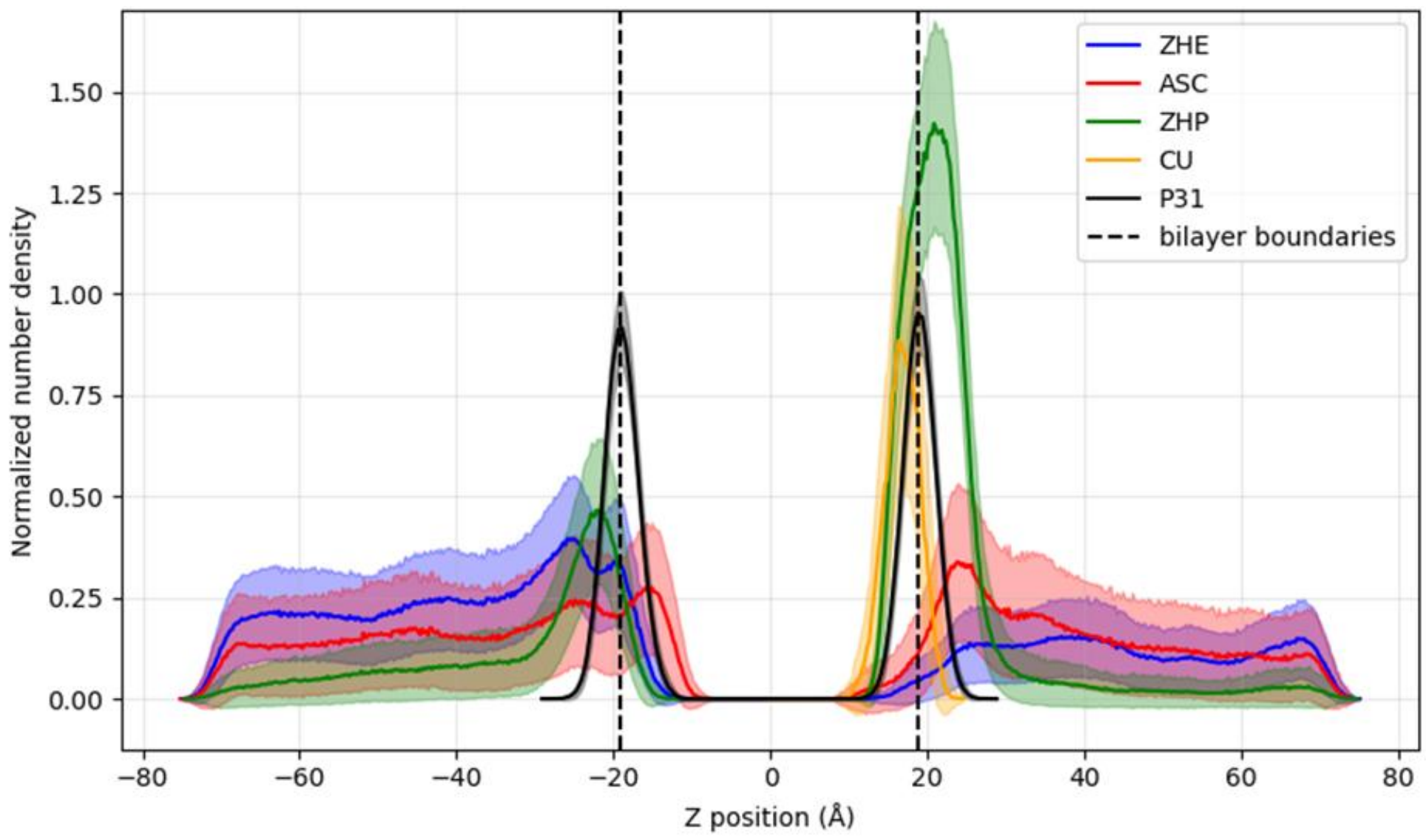

Figure S7: Density profiles made for the last 200 ns of the BBB membrane with the CuNC compounds and averaged for the five replicas. For each species, the average value is shown as a solid line, while the standard deviation limits are shown as a shaded background of the same color. The population values are normalized but, for clarity, the values are multiplied by 10 for ^{31}P and Cu_8. The population peak of the ^{31}P density allows us to locate the membrane boundaries, which are represented by two dotted lines, enabling us to locate the species inside and outside the BBB membrane.

The interaction of CuNC with the lipid membrane does not induce any noticeable perturbation, as evidenced by the ^{31}P density profiles shown in **Figure S6**. These profiles reveal the nearly perfect symmetry of the bilayer, with an identical distribution of the ^{31}P populations in the inner and outer leaflets. Regardless of the replica considered, the position of the ^{31}P atoms remains unchanged, indicating that it is not CuNC that penetrates the membrane but rather the much smaller Cu_8 species.

Analysis of the ligand distributions shown in Figure S6 further reveals distinct localization behaviours for the different solutes. Cu_8 penetrates into the membrane, as its density maximum lies below the ^{31}P peak. In contrast, the other species present in solution (His, His+, and Asc) do not cross the membrane interface but remain beyond the ^{31}P peak, which marks the membrane boundary. Due to electrostatic attraction, His+ tends to localize more prominently near the phosphate groups of the membrane, whereas

ascorbate and neutral histidine display a broader distribution between the polar headgroup region and the solvent.

This behaviour is fully consistent with a vectorization role: the ligands facilitate the approach of Cu_8 to the membrane without directly perturbing it. No translocation of Cu_8 across the membrane is observed within our simulation timescale, which is on the order of microseconds. This likely reflects the limited timescale explored, as full transmembrane diffusion would require longer simulations. Density functional theory (DFT) calculations were performed to further assess the stability of Cu_8 nanoclusters (CuNC) in different solvent environments. Atomization energy variations (ΔE_a) were computed in polar (water) and nonpolar (hexane) media to mimic, respectively, the extracellular aqueous environment and the hydrophobic core of the phospholipid bilayer. The calculated ΔE_a values of -8.76 kcal·mol^{-1} in water and -2.62 kcal·mol^{-1} in hexane indicate that the nanocluster is significantly more stabilized in polar conditions. These results suggest that within the acyl chain region of the membrane, CuNCs are prone to destabilization and partial dissociation into isolated atoms. Consistently, our DFT stability analysis supports that Cu_8 is less stable in a nonpolar medium and may undergo structural distortion within the lipid environment, thereby facilitating its partial embedding into the membrane.

III. Supplementary movies

A. Movies of molecular dynamics

Movies of molecular dynamics are:

- For CuNCs:
  - **Movie 1**: Starting point
  - **Movie 2**: Trajectories
- For associated CuNCs:
  - **Movie 3**: Starting point
  - **Movie 4**: End of analyses

B. Movies of molecular dynamics

Movies of CuNC reaching the BBB membrane by molecular dynamics

- **Movie 5**: Trajectory obtained by MoD simulation with calculations during 100 ns
- **Movie 6**: Trajectory obtained by MoD simulation with calculations started at the end of movie 5 and lasting for 10 µs

C. Movies from the behaviour studies

Typical observation of linear mouse walk test:

- **Movie 7**: Wild-type mice at 28 days
- **Movie 8**: Untreated Mo-blo mice at 28 days
- **Movie 9**: CuNCs treated Mo-blo mice at 28 days

IV. The animal model

Female mice heterozygous for spotted alleles (*Atp7a* Mo-blo) were provided by the Jackson Laboratory (B6Ei.Cg-Atp7aMo-blo/J, strain #002040). These females were mated with pure C57BL/6J strain male mice (strain #000664) with a C57BL/6JEiJ genetic background from Charles River. The hemizygous pups are characterized by a spontaneous mutation of the *atp7a* gene (IVS11 + 3A> C) resulting in copper deficiency, reduced viability and sterility. The mutation of this model is a transversion from A to C in position +3 of the alternate splicing donor of intron 11. Transcriptions derived from this allele altered the splicing mechanism, leading to exon 11 skipping by using a cryptic splicing site further downstream from the correct splicing site. A normal transcription also takes place. Analysis by Western-Blot and enzyme activity showed the production of low levels of non-functional normal-sized proteins. Mo-blo mice have light unspotted coats, are small and have deformed hind legs. These hemizygous males have elastin and collagen defects and die from aortic aneurysm between 3 to 5 weeks of age. They also suffer from pulmonary emphysema related to this same deficit. These mice have brain norepinephrine deprivation, linked to copper deficiency leading to an inactive Dopamine beta-hydroxylase and causing various neurological disorders.

To be more specific, the animals in the providers described allowed the conception of a F1 generation. As the study progressed, crosses between heterozygous females and pure wild males generated mutant male and wild male mice included in the study, but also new heterozygous females to maintain a line of mice available for the study in order to renew the animals necessary for crosses. Precautions have been taken to maintain the previously described phenotype. Indeed, crosses have been maintained with certified pure males in order to avoid genetic drift. In addition, all the mutant mice obtained for the study came from independent mothers for statistical strength. The wild animals included in the study were wild mice from the same crosses as the mutant mice. Sibling animals were defined as a control of mutant mice of the same litter. Moreover, the entire study population was genotyped at 7 days of age to confirm the presence of the mutation. Mo-blo mice possessed a mutation in the *Atp7a* gene characterized by nucleotide transversion at the intron-11 level. This mutation leads to a splicing defect and an exon 11 skipping responsible for the production of a truncated protein.

V. PET analysis

A. Production of 64Cu and radiochemistry

^{64}Cu production was performed using a modified TBP/TK201 column method as previously reported (84). ^{64}Cu was produced on a TR 24 ACSI cyclotron via the $^{64}Ni(p,n)^{64}Cu$ nuclear reaction at a proton energy of 12 MeV and synthesized as $[^{64}Cu]CuCl_2$.

B. Synthesis of ^{64}Cu-histidinate

$^{64}CuCl_2$ in HCl 0.05N (100 MBq) was evaporated to complete dryness. After that, the residue was treated twice with 0.25 ml of water and then evaporated to dryness to ensure the evaporation of all acid residues. Twenty-five µL of $CuCl_2$ (6 mg in 1 ml water) and 75 µL of L-histidine (117.2 mg in 3 ml water) were added, the solution was incubated for 15 min at room temperature. 257 µL of physiological saline solution was added to formulate the ^{64}Cu-histidinate injectable solution. The pH was between 6.3 to 6.7

C. Synthesis of ^{64}CuNCs

To 400 µL of ^{64}Cu-acetate (45 mg in 100 ml water), 600 µL water were added. The solution was transferred in an Eppendorf tube and agitated. Eight mg of histidine were added, and the solution was agitated for further 15 minutes. The solution has turned blue, the pH is adjusted to 12 with 3 drops of NaOH 1M. Twenty-eight mg of ascorbic acid were added under argon, the solution turned pale yellow and was agitated at 40°C for 4 h.

VI. CuNCs effect on lysyl oxidase

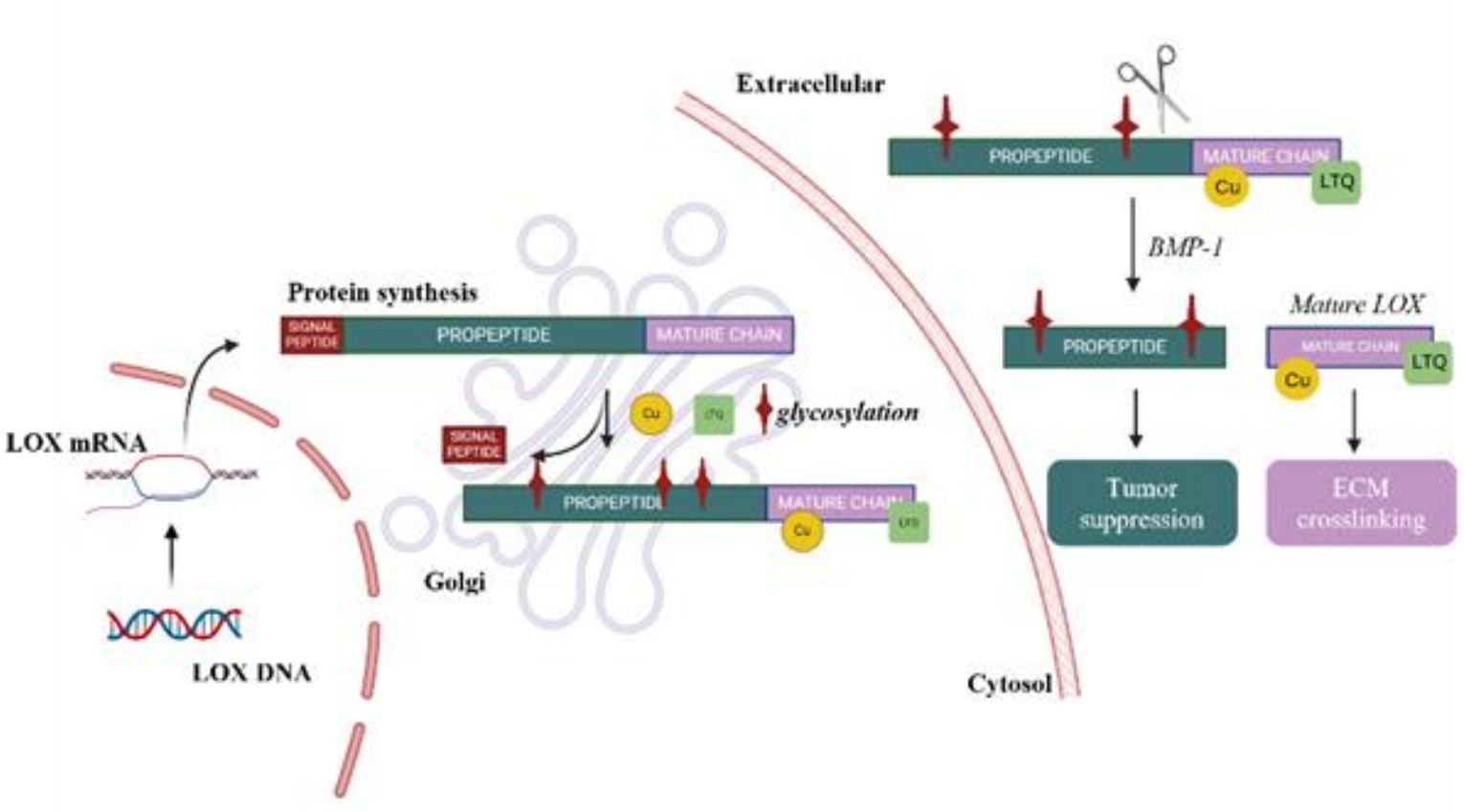

Figure S8: Synthesis and mechanism of action of lysyl oxidase. Transcription and translation of LOX leads to the synthesis of a pre-proLOX form. This pre-proenzyme undergoes post-translational modifications (cleavage of the signal peptide, incorporation of copper, formation of the LTQ cofactor and glycosylation of the pro peptide region) in the endoplasmic reticulum and the Golgi to produce an inactive pro-LOX. This form is released into the extracellular space where it is proteolyzed by pro-collagenases (BMP1) to enable synthesis of the active, mature form of LOX. This protein allows maturation of the ECM and the release of its pro peptide plays a tumour suppressor role (According to (85)). Created in BioRender. Audrey, M. (2026) https://BioRender.com/dxoyufg

Lysyl oxidase is an enzyme involved in collagen synthesis and whose deficiency plays a major role in the survival of animals, which most often die from an aortic or intestinal aneurysms. Its protein expression was studied by WES and Western-blot. Lysyl oxidase is a protein produced in the cell at the reticulum level and then the Golgi where it is glycosylated (inactive precursor) and loaded with copper (Secreted LOX is mature form). Once synthesized and modified, it is secreted to supply target tissues such as the vascular system or all tissues requiring the function of collagen and elastin(85, 86) (ECM crosslinking)(**Fig.S8**). The presence of lysyl oxidase was also studied by Western blotting by differentiating between the glycosylated intracellular form and the mature secreted form.

REFERENCES

1. T. Tønnesen, W. J. Kleijer, N. Horn, Incidence of Menkes disease. *Hum. Genet.* **86**, 408–410 (1991).

2. F. P. E. Vairo, B. C. Chwal, S. Perini, M. A. P. Ferreira, A. C. de Freitas Lopes, J. A. M. Saute, A systematic review and evidence-based guideline for diagnosis and treatment of Menkes disease. *Mol. Genet. Metab.* **126**, 6–13 (2019).

3. H. Kodama, Y. Murata, Molecular genetics and pathophysiology of Menkes disease. *Pediatr. Int.* **41**, 430–435 (1999).

4. M. T. Maung, A. Carlson, M. Olea-Flores, L. Elkhadragy, K. M. Schachtschneider, N. Navarro-Tito, T. Padilla-Benavides, The molecular and cellular basis of copper dysregulation and its relationship with human pathologies. *FASEB J.* **35**, e21810 (2021).

5. N. Horn, T. Tønnesen, Z. Tümer, Menkes disease: An X-linked neurological disorder of the copper metabolism. *Brain Pathol.* **2**, 351–362 (1992).

6. Z. Tümer, L. B. Møller, Menkes disease. *Eur. J. Hum. Genet.* **18**, 511–518 (2010).

7. G. Sherwood, B. Sarkar, A. S. Kortsak, Copper histidinate therapy in Menkes' disease: Prevention of progressive neurodegeneration. *J. Inherit. Metab. Dis.* **12** (Suppl. 2), 393–396 (1989).

8. M. R. Haddad, E.-Y. Choi, P. M. Zerfas, L. Yi, D. Martinelli, P. Sullivan, D. S. Goldstein, J. A. Centeno, L. R. Brinster, M. Ralle, S. G. Kaler, Cerebrospinal fluid-directed rAAV9-rsATP7A plus subcutaneous copper histidinate advance survival and outcomes in a Menkes disease mouse model. *Mol. Ther. Methods Clin. Dev.* **10**, 165–178 (2018).

9. L. Venkataraman, C. J. Jeanty, T. Kaniganti, B. J. Kennedy, P. Sullivan, D. S. Goldstein, S. G. Kaler, Intravenous AAV9-ATP7A plus subcutaneous copper histidinate optimizes outcomes in a lethal Menkes disease mouse model. *Sci. Adv.* **11**, eadw5612 (2025).

10. L. M. Guthrie, S. Soma, S. Yuan, A. Silva, M. Zulkifli, T. C. Snavely, H. F. Greene, E. Nunez, B. Lynch, C. De Ville, V. Shanbhag, F. R. Lopez, A. Acharya, M. J. Petris, B.-E. Kim, V. M. Gohil, J. C. Sacchettini, Elesclomol alleviates Menkes pathology and mortality by escorting Cu to cuproenzymes in mice. *Science* **368**, 620–625 (2020).

11. E. Godoy-Molina, N. L. Serrano, A. Jiménez-González, M. Villaronga, R. M. Marqués Pérez-Bryan, R. Varela-Fernández, S. Lotz-Esquivel, A. H. Tuñón, P. P. Trivedi, N. Horn, J. F. Standing, V. Mangas-Sanjuan, M. Capdevila, A. Mateos, D. Broun, S. Lutsenko, I. Medina-Rivera, R. Artuch, C. Jou, M. Roldán, P. Arango-Sancho, M. Saez-Villafañe, J. J. Ortiz-de-Urbina, A. Pieras-López, M. Duero, R. Farré, J. Pijuan, J. Hoenicka, J. C. Sacchettini, M. J. Petris, V. M. Gohil, F. Palau, Elesclomol-copper therapy improves neurodevelopment in two children with Menkes disease. *J. Clin. Invest.* **135**, e193107 (2025).

12. V. M. Gohil, M. J. Petris, F. Palau, Concerns regarding the safety and efficacy of ES-Cu-Captisol for Menkes disease. Reply. *J. Clin. Invest.* **136**, e202684 (2026).

13. S. G. Kaler, Concerns regarding the safety and efficacy of ES-Cu-Captisol for Menkes disease. *J. Clin. Invest.* **136**, e201900 (2026).

14. Z. Chen, H. Meng, G. Xing, C. Chen, Y. Zhao, G. Jia, T. Wang, H. Yuan, C. Ye, F. Zhao, Z. Chai, C. Zhu, X. Fang, B. Ma, L. Wan, Acute toxicological effects of copper nanoparticles in vivo. *Toxicol. Lett.* **163**, 109–120 (2006).

15. J. F. Hillyer, R. M. Albrecht, Gastrointestinal persorption and tissue distribution of differently sized colloidal gold nanoparticles. *J. Pharm. Sci.* **90**, 1927–1936 (2001).

16. M. Zhu, G. Nie, H. Meng, T. Xia, A. Nel, Y. Zhao, Physicochemical properties determine nanomaterial cellular uptake, transport and fate. *Acc. Chem. Res.* **46**, 622–631 (2013).

17. G. Tosi, J. T. Duskey, J. Kreuter, Nanoparticles as carriers for drug delivery of macromolecules across the blood-brain barrier. *Expert Opin. Drug Deliv.* **17**, 23–32 (2020).

18. M. J. Ernsting, M. Murakami, A. Roy, S.-D. Li, Factors controlling the pharmacokinetics, biodistribution and intratumoral penetration of nanoparticles. *J. Control. Release* **172**, 782–794 (2013).

19. H. Qian, M. Zhu, Z. Wu, R. Jin, Quantum sized gold nanoclusters with atomic precision. *Acc. Chem. Res.* **45**, 1470–1479 (2012).

20. Y. Du, H. Sheng, D. Astruc, M. Zhu, Atomically precise noble metal nanoclusters as efficient catalysts: A bridge between structure and properties. *Chem. Rev.* **120**, 526–622 (2020).

21. X. Song, W. Zhu, X. Ge, R. Li, S. Li, X. Chen, J. Song, J. Xie, X. Chen, H. Yang, A new class of NIR-II gold nanocluster-based protein biolabels for in vivo tumor-targeted imaging. *Angew. Chem. Int. Ed. Engl.* **60**, 1306–1312 (2021).

22. S. Mussa Farkhani, P. Dehghankelishadi, A. Refaat, D. Veerasikku Gopal, A. Cifuentes-Rius, N. H. Voelcker, Tailoring gold nanocluster properties for biomedical applications: From sensing to bioimaging and theranostics. *Prog. Mater. Sci.* **142**, 101229 (2024).

23. Y.-S. Borghei, S. Hosseinkhani, M. R. Ganjali, Bridging from metallic nanoclusters to biomedical in understanding physicochemical interactions at the nano–bio interface. *Part. Part. Syst. Charact.* **39**, 2100202 (2022).

24. J. Wang, P. Li, C. Wang, N. Liu, D. Xing, Molecularly or atomically precise nanostructures for bio-applications: How far have we come? *Mater. Horiz.* **10**, 3304–3324 (2023).

25. A. Boudier, I. Clarot, F. Feillet, Copper Nanoclusters, Methods for obtaining same and the use thereof in the treatment of Menkes disease (2022); https://patentscope.wipo.int/search/en/detail.jsf?docId=WO2022238656.

26. X. Liu, D. Astruc, Atomically precise copper nanoclusters and their applications. *Coord. Chem. Rev.* **359**, 112–126 (2018).

27. B. Starcher, J. A. Madaras, D. Fisk, E. F. Perry, C. H. Hill, Abnormal cellular copper metabolism in the blotchy mouse. *J. Nutr.* **108**, 1229–1233 (1978).

28. J. F. B. Mercer, A. Grimes, L. Ambrosini, P. Lockhart, J. A. Paynter, H. Dierick, T. W. Glover, Mutations in the murine homologue of the Menkes gene in dappled and blotchy mice. *Nat. Genet.* **6**, 374–378 (1994).

29. S. La Fontaine, S. D. Firth, P. J. Lockhart, H. Brooks, J. Camakaris, J. F. Mercer, Intracellular localization and loss of copper responsiveness of Mnk, the murine homologue of the Menkes protein, in cells from blotchy (Mo blo) and brindled (Mo br) mouse mutants. *Hum. Mol. Genet.* **8**, 1069–1075 (1999).

30. M. B. Gawande, A. Goswami, F.-X. Felpin, T. Asefa, X. Huang, R. Silva, X. Zou, R. Zboril, R. S. Varma, Cu and Cu-based nanoparticles: Synthesis and applications in catalysis. *Chem. Rev.* **116**, 3722–3811 (2016).

31. M. Lettieri, P. Palladino, S. Scarano, M. Minunni, Copper nanoclusters and their application for innovative fluorescent detection strategies: An overview. *Sens. Actuators Rep.* **4**, 100108 (2022).

32. W. Wei, Y. Lu, W. Chen, S. Chen, One-pot synthesis, photoluminescence, and electrocatalytic properties of subnanometer-sized copper clusters. *J. Am. Chem. Soc.* **133**, 2060–2063 (2011).

33. A. J. Pell, G. Pintacuda, C. P. Grey, Paramagnetic NMR in solution and the solid state. *Prog. Nucl. Magn. Reson. Spectrosc.* **111**, 1–271 (2019).

34. S. A. Al-thabaiti, A. Y. Obaid, Z. Khan, O. Bashir, S. Hussain, Cu nanoparticles: Synthesis, crystallographic characterization, and stability. *Colloid Polym. Sci.* **293**, 2543–2554 (2015).

35. J. Xiong, Y. Wang, Q. Xue, X. Wu, Synthesis of highly stable dispersions of nanosized copper particles using L-ascorbic acid. *Green Chem.* **13**, 900–904 (2011).

36. J. G. Mesu, T. Visser, F. Soulimani, B. M. Weckhuysen, Infrared and Raman spectroscopic study of pH-induced structural changes of l-histidine in aqueous environment. *Vib. Spectrosc.* **39**, 114–125 (2005).

37. J. Williams, S. Haq, R. Raval, The bonding and orientation of the amino acid l-alanine on Cu{110} determined by RAIRS. *Surf. Sci.* **368**, 303–309 (1996).

38. D. S. Caswell, T. G. Spiro, Ultraviolet resonance Raman spectroscopy of imidazole, histidine, and $Cu(imidazole)_4^{2+}$: Implications for protein studies. *J. Am. Chem. Soc.* **108**, 6470–6477 (1986).

39. M. K. Harbola, Magic numbers for metallic clusters and the principle of maximum hardness. *Proc. Natl. Acad. Sci. U.S.A.* **89**, 1036–1039 (1992).

40. M. Kabir, A. Mookerjee, A. K. Bhattacharya, Copper clusters: Electronic effect dominates over geometric effect. *Eur. Phys. J. D* **31**, 477–485 (2004).

41. M. Criado-Gonzalez, M. I. Peñas, F. Barbault, A. J. Müller, F. Boulmedais, R. Hernández, Salt-induced Fmoc-tripeptide supramolecular hydrogels: A combined experimental and computational study of the self-assembly. *Nanoscale* **16**, 9887–9898 (2024).

42. X. Jia, J. Li, E. Wang, Cu nanoclusters with aggregation induced emission enhancement. *Small* **9**, 3873–3879 (2013).

43. Y. Wang, E. Gallagher, C. Jorgensen, E. P. Troendle, D. Hu, P. C. Searson, M. B. Ulmschneider, An experimentally validated approach to calculate the blood-brain barrier permeability of small molecules. *Sci. Rep.* **9**, 6117 (2019).

44. S. Eragene, J. J. Stewart, J. I. Samuel-Constanzo, T. Tan, N.-Z. Esgdaille, K. J. Bigiarelli, V. D. DaCosta, H. Jimenez, T. R. King, The mouse curly whiskers (*cw*) mutations are recessive alleles of hephaestin-like 1 (*Hephl1*). *Mol. Genet. Metab. Rep.* **20**, 100478 (2019).

45. P. Sharma, M. Reichert, Y. Lu, T. C. Markello, D. R. Adams, P. J. Steinbach, B. K. Fuqua, X. Parisi, S. G. Kaler, C. D. Vulpe, G. J. Anderson, W. A. Gahl, M. C. V. Malicdan, Biallelic HEPHL1 variants impair ferroxidase activity and cause an abnormal hair phenotype. *PLOS Genet.* **15**, e1008143 (2019).

46. N. Horn, P. Wittung-Stafshede, ATP7A-regulated enzyme metalation and trafficking in the Menkes disease puzzle. *Biomedicine* **9**, 391 (2021).

47. C. M. Moore, R. R. Howell, Ectodermal manifestations in Menkes disease. *Clin. Genet.* **28**, 532–540 (1985).

48. A. Jończy, R. Mazgaj, R. R. Starzyński, P. Poznański, M. Szudzik, E. Smuda, M. Kamyczek, P. Lipiński, Relationship between down-regulation of copper-related genes and decreased ferroportin protein level in the duodenum of iron-deficient piglets. *Nutrients* **13**, 104 (2021).

49. S. L. Helman, J. Zhou, B. K. Fuqua, Y. Lu, J. F. Collins, H. Chen, C. D. Vulpe, G. J. Anderson, D. M. Frazer, The biology of mammalian multi-copper ferroxidases. *Biometals* **36**, 263–281 (2023).

50. F. Solano, On the metal cofactor in the tyrosinase family. *Int. J. Mol. Sci.* **19**, 633 (2018).

51. H. Zeng, J. T. Saari, W. T. Johnson, Copper deficiency decreases complex IV but not complex I, II, III, or V in the mitochondrial respiratory chain in rat heart. *J. Nutr.* **137**, 14–18 (2007).

52. J. Gale, E. Aizenman, The physiological and pathophysiological roles of copper in the nervous system. *Eur. J. Neurosci.* **60**, 3505–3543 (2024).

53. S.-K. Cho, S. Gwon, H. A. Kim, J. Kim, S. Y. Cho, D.-E. Kim, J.-H. Chae, D. H. Park, Y. K. Hwang, Abnormal development of neural stem cell niche in the dentate gyrus of Menkes disease. *Int. J. Stem Cells* **15**, 270–282 (2022).

54. Z. Hassan, D. Coelho, T. Kokten, J.-M. Alberto, R. Umoret, J.-L. Daval, J.-L. Guéant, C. Bossenmeyer-Pourié, G. Pourié, Brain susceptibility to methyl donor deficiency: From fetal programming to aging outcome in rats. *Int. J. Mol. Sci.* **20**, 5692 (2019).

55. S. G. Kaler, N. R. Buist, C. S. Holmes, D. S. Goldstein, R. C. Miller, W. A. Gahl, Early copper therapy in classic Menkes disease patients with a novel splicing mutation. *Ann. Neurol.* **38**, 921–928 (1995).

56. EFSA Panel on Dietetic Products, Nutrition and Allergies (NDA), Scientific opinion on dietary reference values for copper. *EFSA J.* **13**, 4253 (2015).

57. S. Ito, S. Del Bino, T. Hirobe, K. Wakamatsu, Improved HPLC conditions to determine eumelanin and pheomelanin contents in biological samples using an ion pair reagent. *Int. J. Mol. Sci.* **21**, 5134 (2020).

58. N. Belcari, N. Camarlinghi, S. Ferretti, P. Iozzo, D. Panetta, P. A. Salvadori, G. Sportelli, A. Del Guerra, NEMA NU-4 performance evaluation of the IRIS PET/CT preclinical scanner. *IEEE Trans. Radiat. Plasma Med. Sci.* **1**, 301–309 (2017).

59. K. Deepika Amirchand, T. S. Banipal, Y.-L. Yang, V. Singh, Volumetric, UV–visible, and computational analysis of molecular interactions between L-ascorbic acid and L-histidine in aqueous solutions. *J. Mol. Liq.* **370**, 120839 (2023).

60. T. Tynkkynen, M. Tiainen, P. Soininen, R. Laatikainen, From proton nuclear magnetic resonance spectra to pH. Assessment of ^{1}H NMR pH indicator compound set for deuterium oxide solutions. *Anal. Chim. Acta* **648**, 105–112 (2009).

61. S. Cikrikci, Benchtop ^{1}H NMR spectroscopy as a practical tool for characterization of chemically ex-changing systems in Ascorbic acid. *J. Adv. Res. Nat. Appl. Sci.* **7**, 35–47 (2021).

62. F. Jofre, M. E. Anderson, J. L. Markley, L-Histidine, Biological Magnetic Resonance Bank (2006); https://doi.org/10.13018/BMSE000039.

63. Q. Cui, I. Lewis, G. Westler, B. Hodis, M. E. Anderson, J. L. Markley, Acetic Acid, Biological Magnetic Resonance Bank (2006); https://doi.org/10.13018/BMSE000191.

64. G. R. Fulmer, A. J. M. Miller, N. H. Sherden, H. E. Gottlieb, A. Nudelman, B. M. Stoltz, J. E. Bercaw, K. I. Goldberg, NMR chemical shifts of trace impurities: common laboratory solvents, organics, and gases in deuterated solvents relevant to the organometallic chemist. *Organometallics* **29**, 2176–2179 (2010).

65. Q. Cui, I. Lewis, G. Westler, M. E. Anderson, J. L. Markley, L-Ascorbate, Biological Magnetic Resonance Bank (2006); https://doi.org/10.13018/BMSE000182.

66. AIST, Spectral Database for Organic Compounds, SDBS. https://sdbs.db.aist.go.jp/CNmrSpectralView.aspx?imgdir=cds&fname=CDS06552&sdbsno=1605.

67. AIST, Spectral Database for Organic Compounds, SDBS. https://sdbs.db.aist.go.jp/CNmrSpectralView.aspx?imgdir=cds&fname=CDS07395&sdbsno=1133.

68. AIST, Spectral Database for Organic Compounds, SDBS. https://sdbs.db.aist.go.jp/CNmrSpectralView.aspx?imgdir=cds&fname=CDS07397&sdbsno=1133.

69. M. J. Frisch, G. W. Trucks, H. B. Schlegel, G. E. Scuseria, M. A. Robb, J. R. Cheeseman, G. Scalmani, V. Barone, G. A. Petersson, H. Nakatsuji, X. Li, M. Caricato, A. V. Marenich, J. Bloino, B. G. Janesko, R. Gomperts, B. Mennucci, H. P. Hratchian, J. V. Ortiz, A. F. Izmaylov, J. L. Sonnenberg, D. Williams-Young, F. Ding, F. Lipparini, F. Egidi, J. Goings, B. Peng, A. Petrone, T. Henderson, D. Ranasinghe, V. G. Zakrzewski, J. Gao, N. Rega, G. Zheng, W. Liang, M. Hada, M. Ehara, K. Toyota, R. Fukuda, J. Hasegawa, M. Ishida, T. Nakajima, Y. Honda, O. Kitao, H. Nakai, T. Vreven, K. Throssell, J. A. Montgomery Jr., J. E. Peralta, F. Ogliaro, M. J. Bearpark, J. J. Heyd, E. N. Brothers, K. N. Kudin, V. N. Staroverov, T. A. Keith, R. Kobayashi, J. Normand, K. Raghavachari, A. P. Rendell, J. C. Burant, S. S. Iyengar, J. Tomasi, M. Cossi, J. M. Millam, M. Klene, C. Adamo, R. Cammi, J. W. Ochterski, R. L. Martin, K. Morokuma, O. Farkas, J. B. Foresman, D. J. Fox, Gaussian 16 Revision C.01 (2016).

70. C. Adamo, V. Barone, Toward reliable density functional methods without adjustable parameters: The PBE0 model. *J. Chem. Phys.* **110**, 6158–6170 (1999).

71. F. Weigend, R. Ahlrichs, Balanced basis sets of split valence, triple zeta valence and quadruple zeta valence quality for H to Rn: Design and assessment of accuracy. *Phys. Chem. Chem. Phys.* **7**, 3297–3305 (2005).

72. D. A. Case, H. M. Aktulga, K. Belfon, I. Y. Ben-Shalom, J. T. Berryman, S. R. Brozell, D. S. Cerutti, T. E. Cheatham, III, G. A. Cisneros, V. W. D. Cruzeiro, T. A. Darden, N. Forouzesh, G. Giambasu, T. Giese, M. K. Gilson, H. Gohlke, A. W. Goetz, R. Harris, S. Izadi, S. A. Izmailov, K. Kasavajhala, M. C. Kaymak, E. King, A. Kovalenko, T. Kurtzman, T. S. Lee, P. Li, C. Lin, J. Liu, T. Luchko, R. Luo, M. Machado, V. Man, M. Manathunga, K. M. Merz, Y. Miao, O. Mikhailovskii, G. Monard, H. Nguyen, K. A. O'Hearn, A. Onufriev, F. Pan, S. Pantano, R. Qi, A. Rahnamoun, D. R. Roe, A. Roitberg, C. Sagui, S. Schott-Verdugo, A. Shajan, J. Shen, C. L. Simmerling, N. R. Skrynnikov, J. Smith, J. Swails, R. C. Walker, J. Wang, J. Wang, H. Wei, X. Wu, Y. Wu, Y. Xiong, Y. Xue, D. M. York, S. Zhao, Q. Zhu, P. A.

Kollman, AMBER24, version 2023 (University of California, San Francisco, 2023); https://ambermd.org/.

73. H. Heinz, T.-J. Lin, R. K. Mishra, F. S. Emami, Thermodynamically consistent force fields for the assembly of inorganic, organic, and biological nanostructures: The INTERFACE force field. *Langmuir* **29**, 1754–1765 (2013).

74. A. H. C. Horn, A consistent force field parameter set for zwitterionic amino acid residues. *J. Mol. Model.* **20**, 2478 (2014).

75. D. Vassetti, M. Pagliai, P. Procacci, Assessment of GAFF2 and OPLS-AA general force fields in combination with the water models TIP3P, SPCE, and OPC3 for the solvation free energy of druglike organic molecules. *J. Chem. Theory Comput.* **15**, 1983–1995 (2019).

76. A. Jakalian, D. B. Jack, C. I. Bayly, Fast, efficient generation of high-quality atomic charges. AM1-BCC model: II. Parameterization and validation. *J. Comput. Chem.* **23**, 1623–1641 (2002).

77. W. L. Jorgensen, J. Chandrasekhar, J. D. Madura, R. W. Impey, M. L. Klein, Comparison of simple potential functions for simulating liquid water. *J. Chem. Phys.* **79**, 926–935 (1983).

78. L. Martínez, R. Andrade, E. G. Birgin, J. M. Martínez, PACKMOL: A package for building initial configurations for molecular dynamics simulations. *J. Comput. Chem.* **30**, 2157–2164 (2009).

79. D. R. Roe, T. E. Cheatham III, PTRAJ and CPPTRAJ: Software for processing and analysis of molecular dynamics trajectory data. *J. Chem. Theory Comput.* **9**, 3084–3095 (2013).

80. W. Humphrey, A. Dalke, K. Schulten, VMD: Visual molecular dynamics. *J. Mol. Graph.* **14**, 33–38 (1996).

81. C. Bannwarth, E. Caldeweyher, S. Ehlert, A. Hansen, P. Pracht, J. Seibert, S. Spicher, S. Grimme, Extendedtight-bindingquantum chemistry methods. *WIREs Comput. Mol. Sci.* **11**, e1493 (2021).

82..A Skjevik, B. D. Madej, R. C. Walker, K. Teigen, LIPID11: A modular framework for lipid simulations using amber. *J. Phys. Chem. B* **116**, 11124–11136 (2012).

83. C. J. Dickson, R. C. Walker, I. R. Gould, Lipid21: Complex lipid membrane simulations with AMBER. *J. Chem. Theory Comput.* **18**, 1726–1736 (2022).

84. J. Svedjehed, C. J. Kutyreff, J. W. Engle, K. Gagnon, Automated, cassette-based isolation and formulation of high-purity [^{61}Cu]$CuCl_2$ from solid Ni targets. *EJNMMI Radiopharm. Chem.* **5**, 21 (2020).

85. C. Rodríguez, J. Martínez-González, The role of lysyl oxidase enzymes in cardiac function and remodeling. *Cells* **8**, 1483 (2019).

86. K. M. Tsang, R. H. Knutsen, C. J. Billington Jr., E. Lindberg, H. Steenbock, Y.-P. Fu, A. Wardlaw-Pickett, D. Liu, D. Malide, Z.-X. Yu, C. K. E. Bleck, J. Brinckmann, B. A. Kozel, Copper-binding domain variation in a novel murine lysyl oxidase model produces structurally inferior aortic elastic fibers whose failure is modified by age, sex, and blood pressure. *Int. J. Mol. Sci.* **23**, 6749 (2022).
